# Therapeutic efficacy of mesenchymal stem cells for abdominal aortic aneurysm: a meta-analysis of preclinical studies

**DOI:** 10.1186/s13287-022-02755-w

**Published:** 2022-02-24

**Authors:** Xintong Li, Hao Wen, Junyuan Lv, Boyang Luan, Jinze Meng, Shiqiang Gong, Jie Wen, Shijie Xin

**Affiliations:** 1 Department of Vascular Surgery, The First Affiliated Hospital of China Medical University, No. 155, Nanjing Street, Heping District, Shenyang, 110001 China; 2 Key Laboratory of Pathogenesis, Prevention and Therapeutics of Aortic Aneurysm in Liaoning Province, Shenyang, China; 3 Department of Trauma Center, The First Affiliated Hospital of China Medical University, Shenyang, China; 4 Department of Breast and Thyroid Surgery, The Affiliated Hospital of Zunyi Medical University, Zunyi, China; 5 Department of Pharmacology, China Medical University, Shenyang, China; 6 Department of Ultrasonography, Inner Mongolia Baotou City Central Hospital, Baotou, China

**Keywords:** Mesenchymal stem cell, Abdominal aortic aneurysm, Meta-analysis, Preclinical studies, Cell therapy

## Abstract

**Background:**

Abdominal aortic aneurysm (AAA) is life-threatening, surgical treatment is currently the only clinically available intervention for the disease. Mesenchymal stem cells (MSCs) have presented eligible immunomodulatory and regenerative abilities which showed favorable therapeutic efficacy in various cardiovascular diseases. However, current evidence summarizing the effectiveness of MSCs for AAA is lacking. Thus, a meta-analysis and systematic review was necessary to be performed to assess the therapeutic efficacy of MSCs for AAA in preclinical studies.

**Methods:**

Comprehensive literature search restricted in English was conducted in PubMed, Cochrane Library, EBSCO, EMBASE and Web of Science from inception to Oct 2021. The primary outcomes were parameters about aortic diameter change during MSCs intervention. The secondary outcomes included elastin content and expression level of inflammatory cytokines, matrix metalloproteinases (MMPs) and their inhibitors (TIMPs). Data were extracted and analyzed independently by two authors. The meta package with random effects model was used to calculate the pooled effect size and 95% confidence intervals in R (version 4.0.2).

**Results:**

Meta-analysis of 18 included studies demonstrated that MSCs intervention has significant therapeutic effects on suppressing aortic diameter enlargement compared with the control group (diameter, SMD = − 1.19, 95% CI [− 1.47, − 0.91]; diameter change ratio, SMD = − 1.36, 95% CI [− 1.72, − 1.00]). Subgroup analysis revealed differences between MSCs and control group regarding to cell type, intervention route and cell compatibility. Moreover, the meta-analysis also showed that MSCs intervention had a significant effect on preserving aortic elastin content, reducing MCP-1, TNF-α, IL-6, MMP-2/9 and increasing TIMP-1/2 expression level compared with control group.

**Conclusion:**

Our results suggested that MSC intervention is effective in AAA by suppressing aortic diameter enlargement, reducing elastin degradation, and modulating local immunoinflammatory reactions. These results are important for the systemic application of MSCs as a potential treatment candidate for AAA in further animal experiments and clinical trials.

**Supplementary Information:**

The online version contains supplementary material available at 10.1186/s13287-022-02755-w.

## Introduction

Abdominal aortic aneurysm (AAA) is permanent pathologic dilatation of the abdominal aorta, of which the maximum diameter of lesion area is 1.5 folds greater than the normal segment or more than 3 cm regardless of differences in patient gender and stature [1, 2]. In recent years, the level of clinical treatment of AAA has been improving with the continuous advancements of endovascular treatment techniques [3]. However, to date, no effective drugs or non-surgical therapies have been developed [4]. It is also expected that the reliable medical treatment will inevitably slow disease progression and improve clinical prognosis, until the current indications for surgical intervention are met. Therefore, the exploration of non-surgical treatment is of great significance for the clinical intervention of AAA.

The pathogenesis of AAA mainly includes infiltration of inflammatory cells such as macrophages, lymphocytes and neutrophils, degradation of the extracellular matrix mediated by matrix metalloproteinases, as well as apoptosis and phenotypic transition of medial vascular smooth muscle cells (VSMCs) [5, 6]. Cellular therapies, especially those based on mesenchymal stem cells (MSCs), have recently demonstrated inspiring repair capabilities in diseases such as spinal cord injury [7], cardiovascular diseases [8–10], and Crohn's disease [11, 12]. MSCs were reported to exert reparative capabilities by secreting various cytokines or exosomes to modulate local inflammatory reactions and mediate intercellular communications, and being capable of migrating to the lesion sites and differentiating into functional cells [13, 14].

Preclinical studies have been conducted to investigate the mechanisms and efficacy of MSCs intervention in AAA. For example, Sharma et al. [15] proved that experimental AAA was attenuated by human placenta derived MSCs. Moreover, our previous study found intravenous injection with human umbilical cord derived MSCs could halt aneurysm enlargement, suppress elastin degradation, inhibit MMP-2/9 and TNF-α expression, and preserve/restore VSMC contractile phenotype [16]. Although results of preclinical studies are rigorous and inspiring, given the limitations of individual studies and the heterogeneity among studies, a pooled analysis about the overall therapeutic effects of MSCs for AAA in preclinical studies is necessary.

Therefore, this systematic review and meta-analysis was implemented to assess the efficacy of MSCs treatment in animals with AAA. Our findings will provide a theoretical basis and guide the clinical application of MSCs-based therapy for AAA.

## Methods

The implementation of this systematic review followed the guiding principles presented in the Preferred Reporting Items for Systematic Reviews and Meta-analyses (PRISMA) criteria [17]. The details about PRISMA checklist for this meta-analysis is presented in Additional file 1. In addition, the protocol for this systematic review was registered on PROSPERO (registered ID: CRD42020218430) and can be accessed at www.crd.york.ac.uk/PROSPERO/display_record.asp?ID = CRD42020218430.

### Literature search strategy

We conducted a comprehensive search process to evaluate the therapeutic efficacy of MSCs therapy for AAA in preclinical studies. Literatures in PubMed, EMBASE, Cochrane Library, EBSCO and Web of Science databases were searched from start to Oct 2021 by two independent authors (HW and JW). The keywords used in the search process include “stem cell*”, “stromal cell*”, “cell transplantation”, “progenitor cell*”, “precusor cell*”, “cell* therap*” and combination with “abdominal aortic aneurysm*”, the detailed search strategy was presented in Additional file 2. In addition, we also conducted manual search for references of relevant reviews and included studies eventually. All literatures retrieved from the above databases and manual retrieval process were imported into Endnote (version X9.3.3), a literature management software, for identifying and removing duplicates, conference abstracts, reviews and irrelevant articles.

### Inclusion and exclusion criteria

Eligible preclinical studies with outcomes including assessment of the efficacy of MSCs therapy in AAA were included. Studies should be originally published in peer-reviewed journals in English. The primary outcomes for inclusion in this review was the absolute final value (mm) or change ratio (% increase) of maximum aortic diameter following MSCs therapy. Secondary outcomes included elastin content, changes in cytokines expression levels and indicators of inflammatory responses. All articles reporting primary outcomes were included, regardless of whether secondary outcomes were reported. Exclusion criteria for this study were non-animal studies, non-MSC intervention, not AAA animal model, in-vitro studies, the experimental group did not receive stem cell therapy or was not simply receiving stem cell therapy alone, and the reported outcomes did not include indicators of maximum aortic diameter.

### Data extraction

The process of data extraction in this study was carried out by two independent researchers (JYL and HW). For data extraction with disagreement, the decision was made by a third researcher (SQG) or after group discussion, and the results were summarized afterwards. Following the carefully conducted data extraction, we summarized and documented the details of each study including author, year, country, and details of animal models induction including animal species, sex, and modeling method, as well as details of intervention including cell type, compatibility, route, frequency, total dose, intervention duration (defined as time duration from initial cell intervention to sacrifice). Data at different time points during the follow-up period of the same study were included for analysis. If data results were presented in picture form only, we attempted to obtain data by contacting the corresponding authors, otherwise, the WebPlotDigitizer [18] software was implemented for the measurements of continuous values.

### Assessment of risk of bias

The risk of bias (RoB) in this review was evaluated following the principles from the Systematic Review Centre for Laboratory Animal Experimentation (SYRCLE) RoB tool [19]. The risk of bias was assessed by two independent authors (JZM and BYL) and disagreement was settled by the third author (SQG). Terms of risk of bias in this review includes selection bias (1. sequence generation, 2. baseline characteristics, 3. allocation concealment), performance bias (4. random housing, 5. blinding of investigators), detection bias (6. random animals assessment, 7. blinding of outcome assessor), attrition bias (8. incomplete outcome data), reporting bias (9. selective outcome reporting), and 10. other sources of bias. For each included study, the risk of bias was scored as high, low, or unclear.

### Statistical analysis

Data analysis in this study were performed using meta package (version 4.18-2) [20] in R software (version 4.0.2). The results of continuous variables were expressed using standard mean difference (SMD) with the 95% confidence interval (CI). SMD were calculated by the mean of outcome, standard deviation (SD), and case number of different arms in each study. The pooled SMD was processed using the random effects model to generate forest plot. Between-study heterogeneity were analyzed by the Q test and *I*^2^ statistics. *I*^2^ < 40% with *P* > 0.05 represents low heterogeneity, 30% to 60% with *P* < 0.05 represents moderate heterogeneity, 50% to 90% represents substantial heterogeneity, 75% to 100% represents considerable heterogeneity. Subgroup analysis was implemented to identify potential source of heterogeneity and explore other influencing factors. Differences with a two-tailed *P* < 0.05 were considered statistically significant.

## Results

### Study selection

**Fig. 1:**
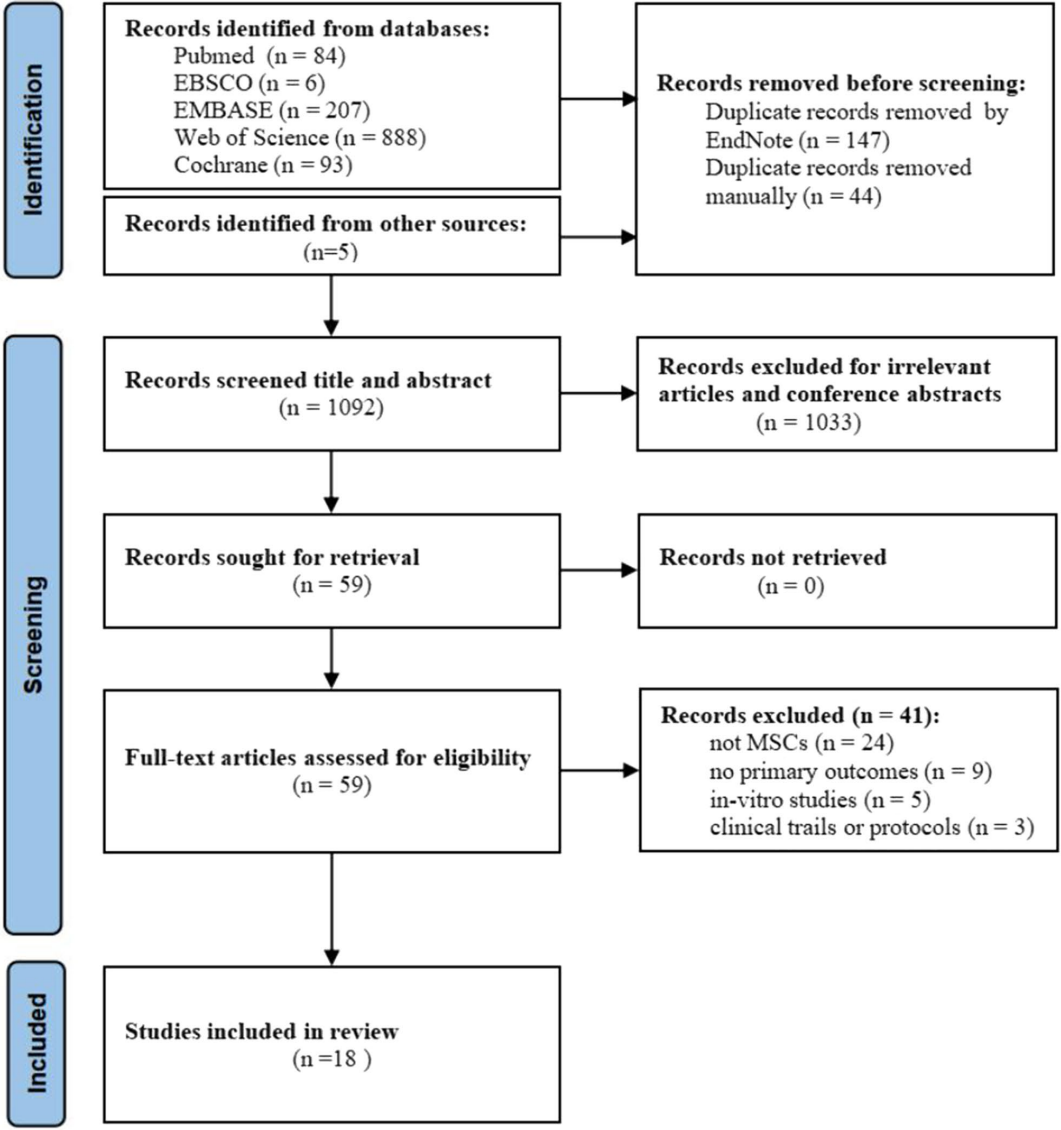
Flow diagram for study search, screening and selection

According to the results of literature search, a total of 1283 articles were identified and 1092 were retained after duplicates removed. Next, after reviewing the titles and abstracts of all the articles, 59 articles were isolated and assessed for full-text review. Ultimately, 18 studies were included in this meta-analysis. The detailed study selection process was illustrated in Fig. 1.

### Study characteristics

Detailed characteristics of the included studies are summarized in Table 1. Studies were published from 2011 to 2021, with 6 studies conducted in USA [15, 21–25], 6 studies in Japan [26–31], 3 studies in France [32–34], 2 studies in China [16, 35] and 1 study in Netherlands [36]. For AAA model induction, 7 studies established AAA model with elastase [15, 16, 21–25], 6 studies with Ang II [16, 27, 28, 30, 31, 35], 3 studies with xenograft techniques [32–34] and 2 studies were established with the combination of elastase and CaCl_2_ [29, 36]. For animal species, 13 studies were implemented with mice including 6 studies with ApoE-/- mice [26–28, 30, 31, 35], 6 studies with C57BL/6 mice [15, 21–25] and 1 study with SCID mice [29], 5 studies were implemented with rats including 4 studies with Fisher rats [32–34, 36] and 1 study with SD rats [16]. In terms of cell types, 11 studies used bone marrow derived MSCs [22, 26–35], while 3 studies used adipose derived MSCs [21, 25, 36], 3 studies used umbilical cord derived MSCs [16, 23, 24] and only 1 study used placenta derived MSCs [15]. In regard to cell compatibility, 12 studies utilized allogenic cells isolated from the same species [21, 22, 26–28, 30–36], while 6 studies were xenogeneic [15, 16, 23–25, 29] since cells were isolated from human donors. For intervention route, 12 studies infused MSCs by intravenous injection [15, 16, 22–27, 29–31, 35], 3 studies by perivascular incubation [21, 28, 36] and 3 studies applied with intraluminal incubation [32–34]. Intervention frequencies ranged from 1 to 4 times, and the total cell dose ranged from 6*10^4^ cells to 9*10^6^ cells. In terms of primary outcomes, 13 studies reported results of final value (mm) of maximal aortic diameter [16, 21, 25–31, 33–36] and 9 studies reported change ratio of diameter (% increase) [15, 16, 22–24, 29, 32–34].

**Table 1:** Characteristics of preclinical studies investigating the therapeutic efficacy of MSCs in AAA models

Author	Year	Country	No. of MSC group	No. of control group	AAA model	Animal species	MSC source	Cell compatibility	Intervention route	Total dose (cells)	Control	Follow-up duration (days)
Hashizume [28]	2011	Japan	7	6	AngII	apoE^−/−^ mice	Bone marrow	Allogenic	Perivascular	1*10^5^	NS	28
Sharma [15]	2012	USA	6	8	Elastase	C57BL/6 mice	Placenta	Xenogenic	Intravenous	1*10^6^	NS	14
Fu-1 [27]	2013	Japan	10	5	AngII	apoE^−/−^ mice	Bone marrow	Allogenic	Intravenous	1*10^6^	Saline	28
Fu-2 [27]	2013	Japan	12	5	AngII	apoE^−/−^ mice	Bone marrow	Allogenic	Intravenous	4*10^6^	Saline	28
Schneider-1 [32]	2013	France	6	3	Xenograft	Fischer rats	Bone marrow	Allogenic	Intraluminal	1*10^6^	medium	7
Schneider-2 [32]	2013	France	5	3	Xenograft	Fischer rats	Bone marrow	Allogenic	Intraluminal	1*10^6^	medium	28
Blose [21]	2014	USA	7	6	Elastase	C57BL/6 mice	Adipose tissue	Allogenic	Perivascular	1*10^5^	Saline	9
Yamawaki-Ogata-1 [30]	2014	Japan	10	10	AngII	apoE^−/−^ mice	Bone marrow	Allogenic	Intravenous	1*10^6^	Saline	14
Yamawaki-Ogata-2 [30]	2014	Japan	7	6	AngII	apoE^−/−^ mice	Bone marrow	Allogenic	Intravenous	1*10^6^	Saline	28
Yamawaki-Ogata-3 [30]	2014	Japan	6	5	AngII	apoE^−/−^ mice	Bone marrow	Allogenic	Intravenous	1*10^6^	Saline	56
Zidi [33]	2015	France	6	6	Xenograft	Fischer rats	Bone marrow	Allogenic	Intraluminal	1*10^6^	NS	7
Davis-1 [22]	2015	USA	11	4	Elastase	C57BL/6 mice	Bone marrow	Allogenic	Intravenous	9 × 10^6^	NS	14
Davis-2 [22]	2015	USA	9	4	Elastase	C57BL/6 mice	Bone marrow	Allogenic	Intravenous	9 × 10^6^	NS	14
Sharma [23]	2016	USA	8	8	Elastase	C57BL/6 mice	Umbilical cord	Xenogenic	Intravenous	1*10^6^	NS	14
Xie-1 [25]	2017	USA	4	4	Elastase	C57BL/6 mice	Adipose tissue	Xenogenic	Intravenous	1*10^6^	PBS	4
Xie-2 [25]	2017	USA	5	5	Elastase	C57BL/6 mice	Adipose tissue	Xenogenic	Intravenous	1*10^6^	PBS	7
Xie-3 [25]	2017	USA	4	4	Elastase	C57BL/6 mice	Adipose tissue	Xenogenic	Intravenous	1*10^6^	PBS	14
Yamawaki-Ogata [31]	2017	Japan	5	5	AngII	apoE^−/−^ mice	Bone marrow	Allogenic	Intravenous	1*10^6^	Saline	14
Hosoyama-1 [29]	2018	Japan	24	8	Elastase CaCl2	SCID mice	Bone marrow	Xenogenic	Intravenous	6*10^4^	PBS	7, 14, 21
Hosoyama-2 [29]	2018	Japan	24	8	Elastase CaCl2	SCID mice	Bone marrow	Xenogenic	Intravenous	6*10^4^	PBS	28, 35, 42, 56, 64
Parvizi [36]	2018	Netherlands	6	6	Elastase CaCl2	Fischer rats	Adipose tissue	Allogenic	Perivascular	2*10^6^	NS	14
Spinosa [24]	2018	USA	12	12	Elastase	C57BL/6 mice	Umbilical cord	Xenogenic	Intravenous	1*10^6^	NS	14
Zidi [34]	2018	France	6	6	Xenograft	Fischer rats	Bone marrow	Allogenic	Intraluminal	1*10^6^	NS	7
Zhou [35]	2019	China	8	8	AngII	apoE^−/−^ mice	Bone marrow	Allogenic	Intravenous	2*10^6^	medium	14
Wen-1 [16]	2020	China	10	5	Elastase	SD rats	Umbilical cord	Xenogenic	Intravenous	1*10^6^	Saline	7
Wen-2 [16]	2020	China	10	5	Elastase	SD rats	Umbilical cord	Xenogenic	Intravenous	1*10^6^	Saline	14
Akita-1 [26]	2021	Japan	10	5	AngII	apoE^−/−^ mice	Bone marrow	Allogenic	Intravenous	1*10^6^	Saline	14
Akita-2 [26]	2021	Japan	10	5	AngII	apoE^−/−^ mice	Bone marrow	Allogenic	Intravenous	1*10^6^	Saline	14

MSCs, mesenchymal stem cells; AAA, abdominal aortic aneurysm; AngII, angiotensin II; apoE^−/−^, apolipoprotein E knockout mice; PBS, phosphate buffered saline; NS, not mentioned

### Risk of bias

The assessment of Rob was summarized in Table 2. Almost all studies mentioned randomized grouping, but none specified the method. All included studies showed similar features in terms of the baseline characteristics, which reduced the risk of selection bias. No study properly described the method of allocation concealment, performance blinding and detection blinding. Two studies [21, 25] were considered to have attrition bias because the reasons for missing samples were not reported. In addition, we did not identify any additional sources of bias.

**Table 2:** SYRCLE risk of bias assessment of included studies

Author (year)	A	B	C	D	E	F	G	H	I	J
Hashizume (2011) [28]	Y	Y	U	Y	U	U	U	Y	Y	U
Sharma (2012) [15]	Y	Y	U	Y	U	U	U	Y	Y	U
Fu (2013) [27]	Y	Y	U	Y	U	U	U	Y	Y	U
Schneider (2013) [32]	Y	Y	U	Y	U	U	U	Y	Y	U
Blose (2014) [21]	U	Y	U	Y	U	U	U	N	Y	U
Yamawaki-Ogata (2014) [30]	Y	Y	U	Y	U	U	U	Y	Y	U
Zidi (2014) [33]	U	Y	U	U	U	U	U	Y	Y	U
Davis (2015) [22]	Y	Y	U	Y	U	U	U	Y	Y	U
Sharma (2016) [23]	Y	Y	U	Y	U	U	U	Y	Y	U
Xie (2017) [25]	U	Y	U	Y	U	U	U	N	U	U
Yamawaki-Ogata (2017) [31]	Y	Y	U	Y	U	U	U	Y	Y	U
Hosoyama (2018) [29]	Y	Y	U	Y	U	U	U	Y	Y	U
Parvizi (2018) [36]	Y	Y	U	U	U	U	U	Y	Y	U
Spinosa (2018) [24]	Y	Y	U	Y	U	U	U	Y	Y	U
Zidi (2018) [34]	U	Y	U	U	U	U	U	Y	Y	U
Zhou (2019) [35]	Y	Y	U	Y	U	U	U	Y	Y	U
Wen (2020) [16]	Y	Y	U	Y	U	U	U	Y	Y	U
Akita (2021) [26]	Y	Y	U	U	U	U	U	Y	Y	U

A: Sequence generation; B: Baseline characteristics; C: Allocation concealment; D: Random housing; E: Performance blinding; F: Random outcome assessment; G: Detection blinding; H: Incomplete outcome data; I: Selective outcome reporting; J: Other sources of bias

### Primary outcomes

#### Maximum aortic diameter

**Fig. 2:**
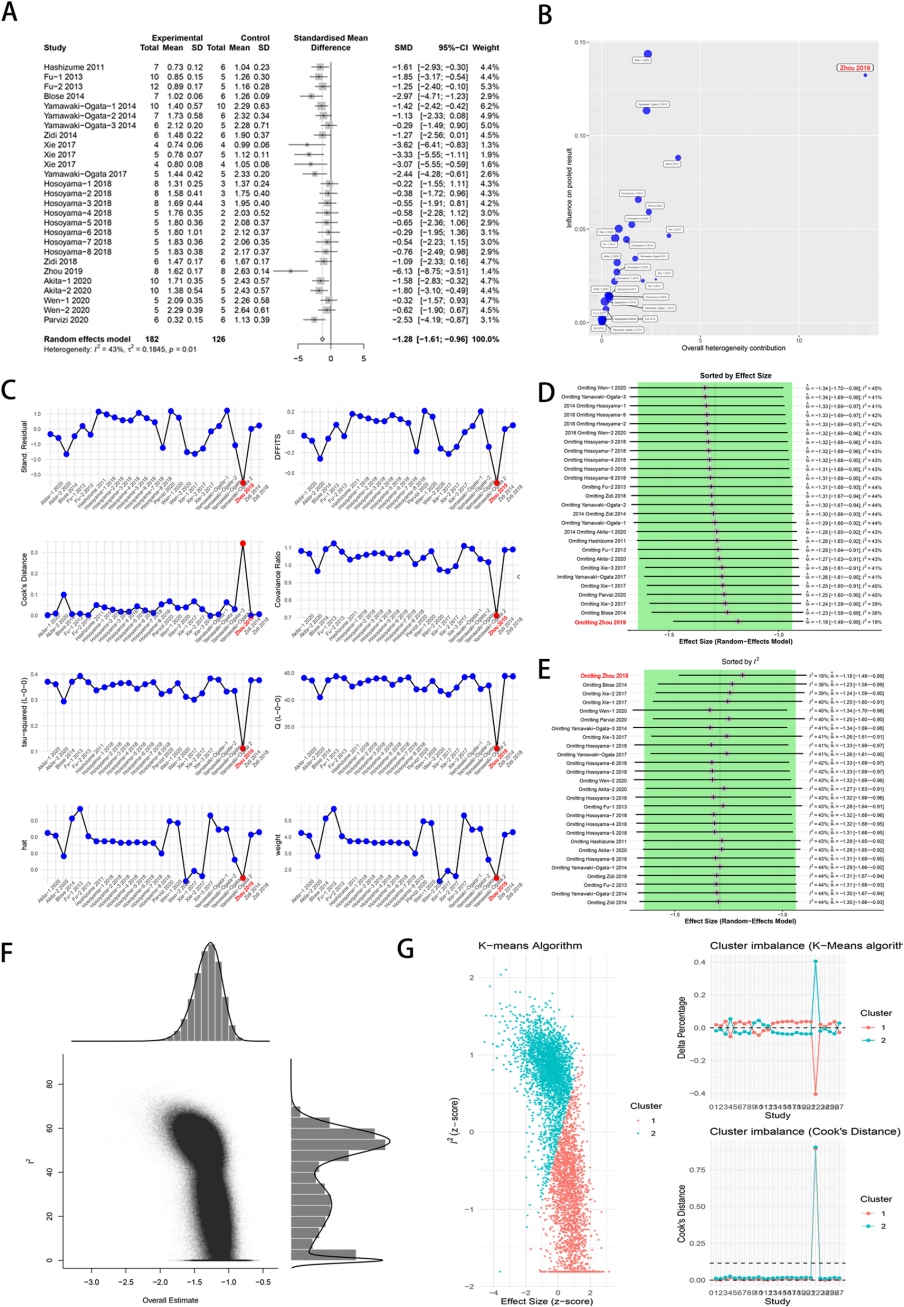
Identification of outlier study regarding to maximum aortic diameter. **A** The original forest plot. **B** Baujat plot. **C** Influence diagnostics. **D**, **E** Leave-one-out meta-analysis ranked by effect size and *I*^2^, respectively. **F**, **G** GOSH and GOSH diagnostic (k-means algorithm) plots, respectively. SMD: standard mean difference; 95% CI, 95% confidence interval

**Fig. 3:**
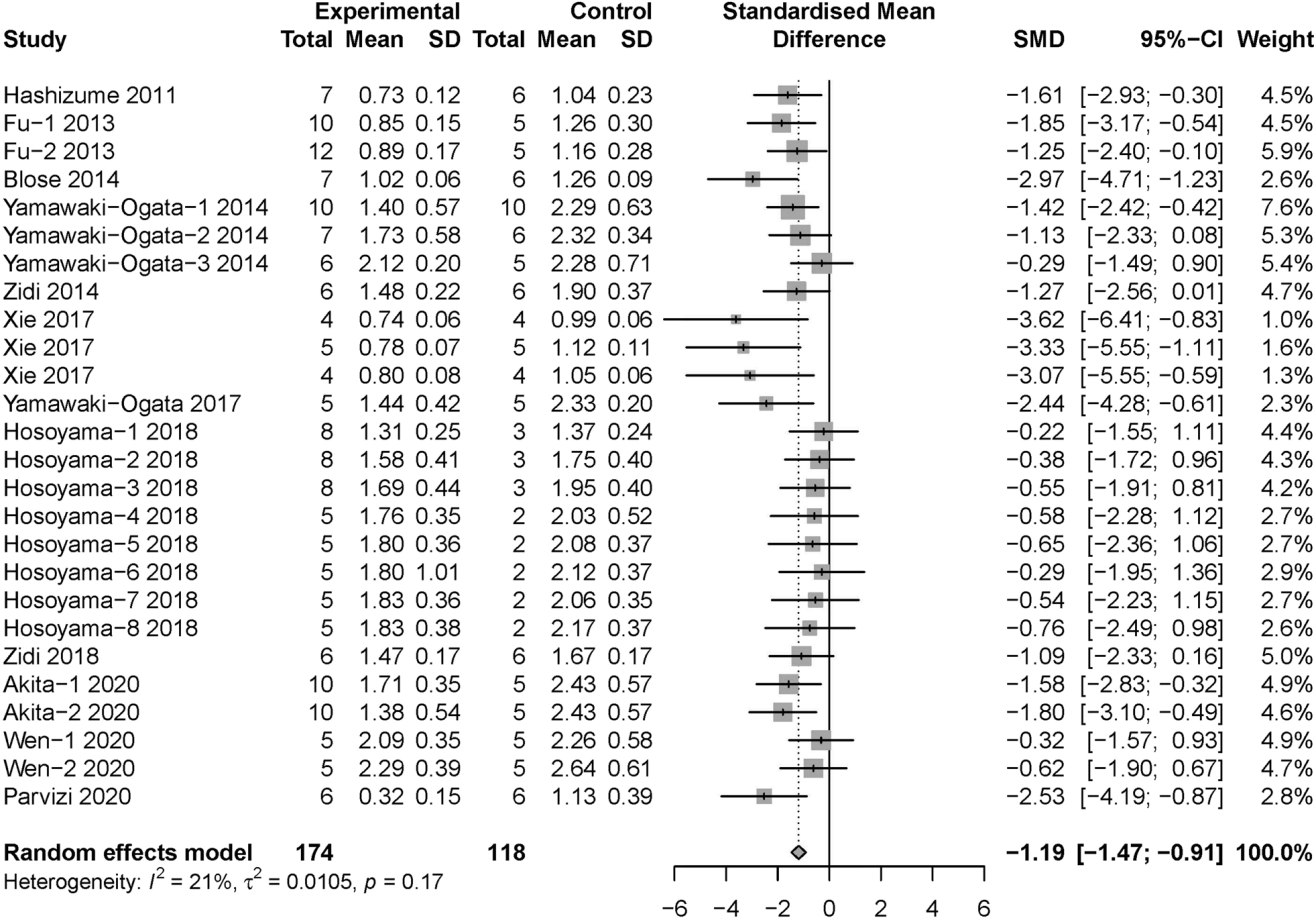
The forest plot: the therapeutic effects of MSCs for maximum aortic diameter in AAA models, compared with control group

A total of 13 original studies including 27 experimental arms reported the final value of maximum aortic diameter in the animal model of AAA after MSCs intervention [16, 21, 25–31, 33–36]. Meta analysis with random effects model showed that MSCs intervention significantly reduced the final value of maximum diameter compared with the control group (SMD = − 1.28, 95% CI [− 1.61, − 0.96]; *P* < 0.05; Fig. 2A). However, a moderate between-study heterogeneity (*I*^2^ = 43%, *P* = 0.01) was identified. Thus, Baujat plot [37], influence diagnostics [38], leave-one-out meta-analysis and GOSH plot analysis [39] were conducted to determine the outlier studies. Baujat plot revealed that the study by Zhou et al. [35] contributes the most to the overall heterogeneity (Fig. 2B). Influence diagnostics analysis revealed that the study by Zhou et al. [35] could have distorted our pooled effect estimate and partially contributed to the between-study heterogeneity we found in our initial meta-analysis (Fig. 2C). Leave-one-out meta-analysis recalculated pooled effect, with one study omitted each time, which generated two forest plots ordered by recalculated effect size and *I*^2^, respectively. We found that the overall effect is narrowed when Zhou’s study [35] was omitted and yielded the smallest heterogeneity (Fig. 2D, E). Moreover, GOSH plot analysis also identified the study by Zhou et al. [35]as the main contributor to the overall heterogeneity (Fig. 2F, G). Thus, the meta-analysis was reconducted with Zhou’s study omitted, results showed a smaller but significant overall effect size (SMD = − 1.19, 95% CI [− 1.47, − 0.91]; *P* < 0.05) with low between-study heterogeneity (*I*^2^ = 21%, *P* = 0.17) (Fig. 3).

**Fig. 4:**
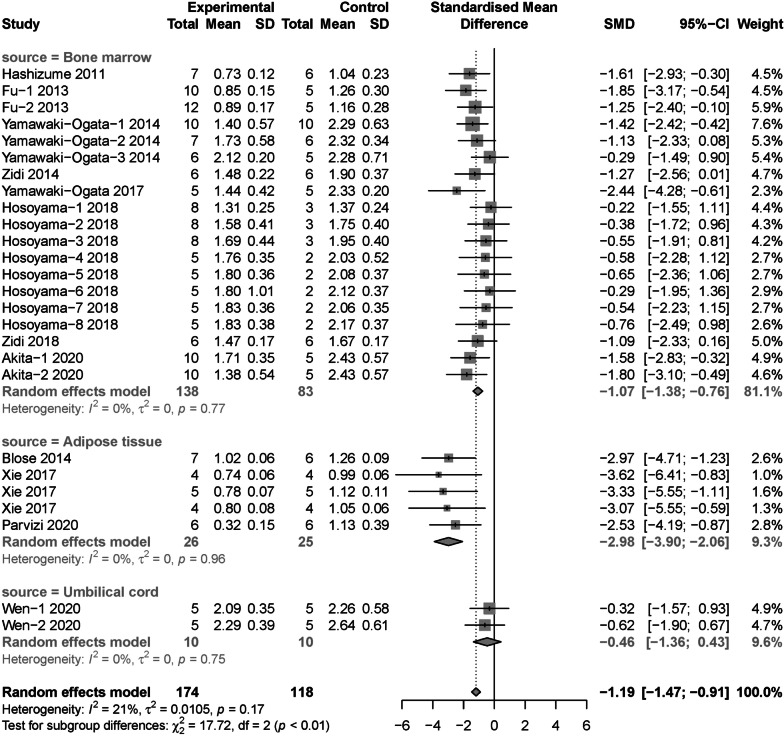
Subgroup analysis: the different therapeutic effects of MSCs for maximum aortic diameter in AAA models regarding to cell source

**Fig. 5:**
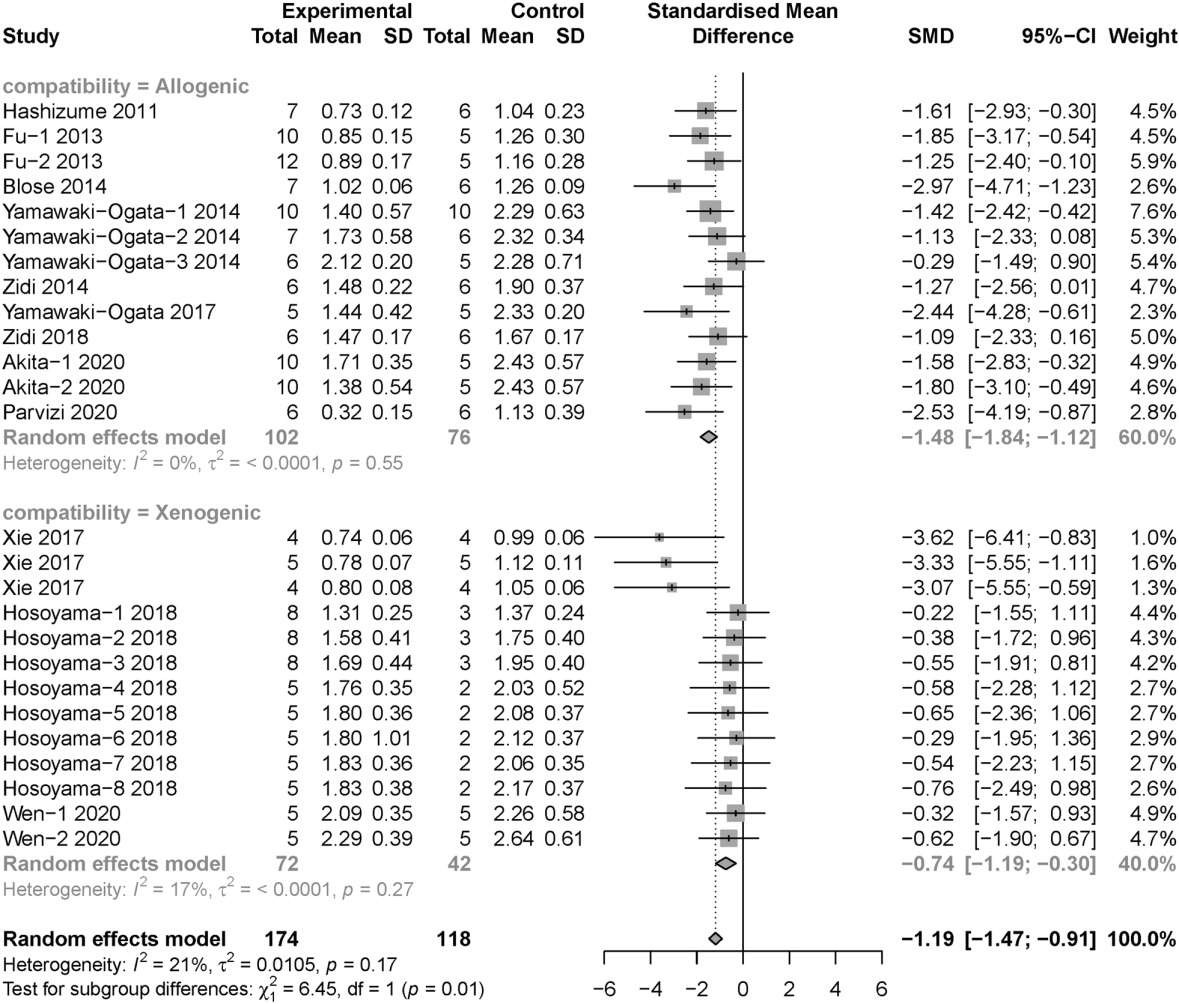
Subgroup analysis: the different therapeutic effects of MSCs for maximum aortic diameter in AAA models regarding to cell compatibility

Subgroup analysis regarded to maximum aortic diameter were conducted with Zhou’s study omitted. In terms of cell source, adipose tissue derived MSCs (SMD = − 2.98, 95% CI [− 3.90, − 2.06]) showed significant decrease in diameter than MSCs from bone marrow (SMD = − 1.07, 95% CI [− 1.38, − 0.76]) and umbilical cord (SMD = − 0.46, 95% CI [− 1.36, 0.43]) (*P* < 0.01) (Fig. 4). For cell compatibility, allogenic MSCs (SMD = − 1.48, 95% CI [− 1.84, − 1.12]) was more effective than xenogeneic MSCs (SMD = − 0.74, 95% CI [− 1.19, − 0.30]) (*P* = 0.02) (Fig. 5). In regard to intervention route, perivascular incubation (SMD = − 2.23, 95% CI [− 3.11, − 1.34]) seemed to be more efficient than intravenous injection (SMD = − 1.06, 95% CI [− 1.37, − 0.75]) and intraluminal incubation (SMD = − 1.18, 95% CI [− 2.07, − 0.28]), although not statistically significant (*P* = 0.05) (Additional file 3: Fig. S1). For total cell dose, ≥ 1*10^6^ cells group (SMD = − 1.37, 95% CI [− 1.74, − 1.03]) seemed to be more efficient than < 1*10^6^ cells group (SMD = − 0.82, 95% CI [− 1.30, − 0.34]), although still not statistically significant (*P* = 0.07) (Additional file 4: Fig. S2). Moreover, the pooled effect size at different follow-up duration groups was not statistical significant (*P* = 0.06), given the ≤ 2 week group (SMD = − 1.47, 95% CI [− 1.95, − 1.00]) was higher than ≤ 4 weeks group (SMD = − 1.21, 95% CI [− 1.75, − 0.68]), the result of > 4 weeks group was not significant (SMD = − 0.47, 95% CI [− 1.16, 0.23]) (Additional file 5: Fig. S3). Subgroup analysis for animal model induction methods (Additional file 6: Fig. S4), animal species (Additional file 7: Fig. S5) and control type (Additional file 8: Fig. S6) were not statistically significant.

#### Maximum aortic diameter change ratio (% increase)

**Fig. 6:**
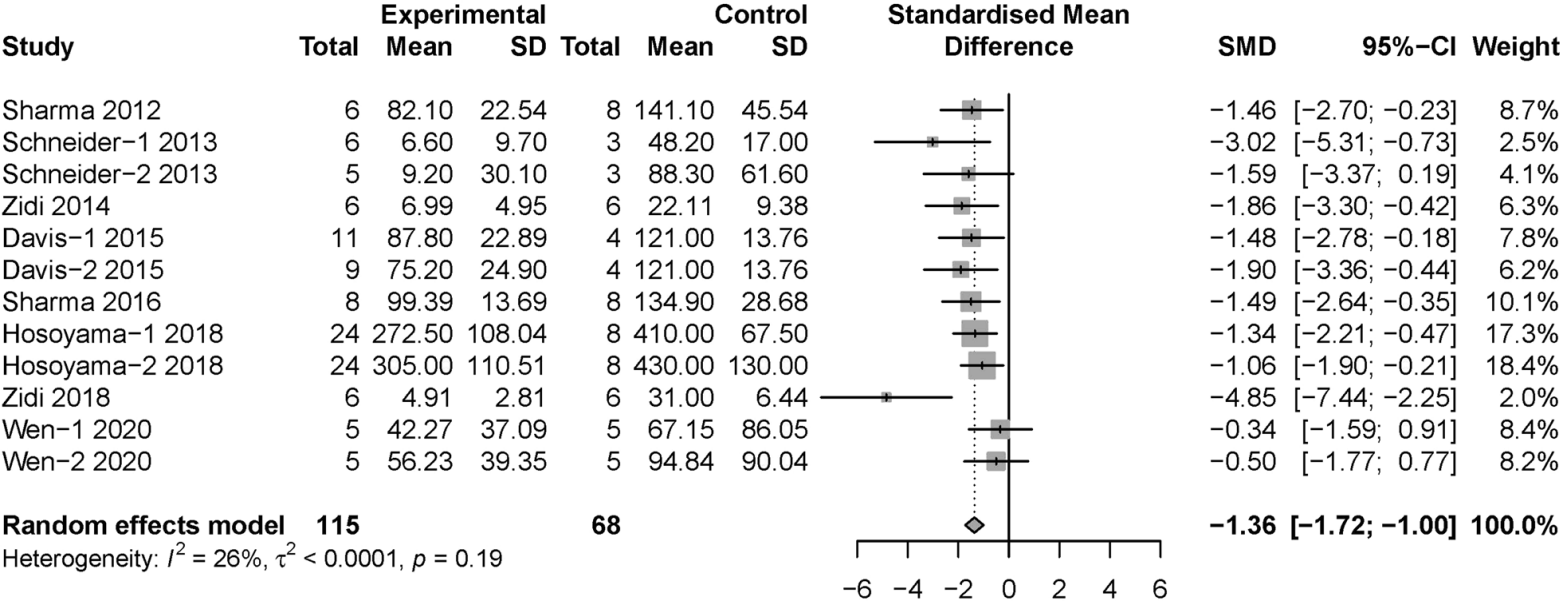
The forest plot: the therapeutic effects of MSCs for change ratio of maximum aortic diameter in AAA models, compared with control group

In our study, a total of 9 articles including 13 experimental arms reported data about change ratio of maximum aortic diameter [15, 16, 22–24, 29, 32–34]. Meta-analysis based on random effects model showed that MSCs intervention reduced change ratio of maximum aortic diameter compared with control group in AAA (SMD = − 1.58, 95% CI [− 2.05, − 1.11]), moderate between-study heterogeneity (*I*^2^ = 45%, *P* = 0.04) was identified (Additional file 9: Fig. S7). The study by Spinosa et al. [24] was identified as the major contributor to the overall heterogeneity (Additional file 10: Fig. S8). Thus, after omitting Spinosa’s study [24], pooled effect was recalculated with random effects model. Results showed MSCs treatment could reduce change ratio of maximal aortic diameter in AAA model (SMD = − 1.36, 95% CI [− 1.72, − 1.00]) with low between-study heterogeneity (*I*^2^ = 26%, *P* = 0.19) (Fig. 6).

**Fig. 7:**
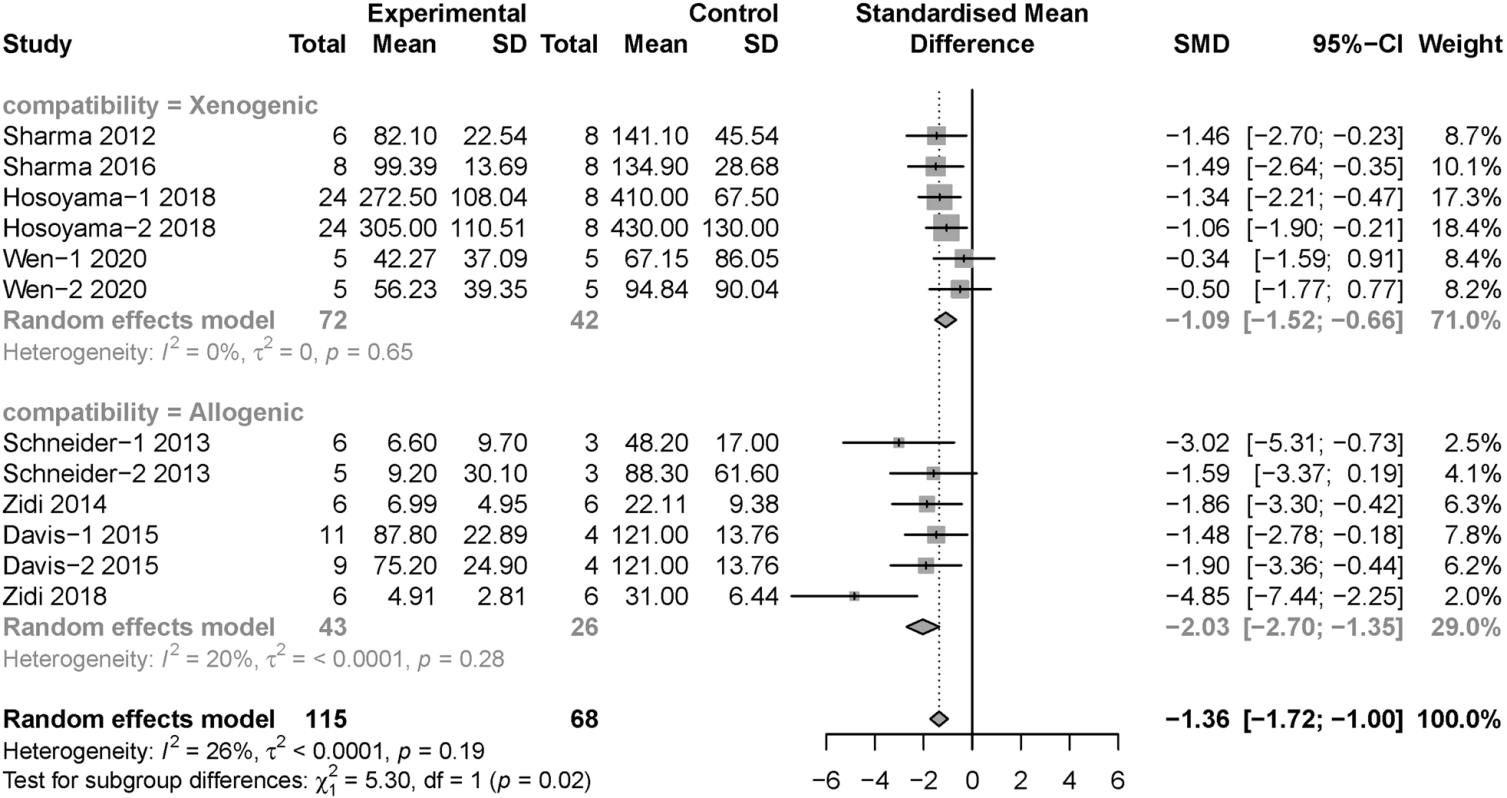
Subgroup analysis: the different therapeutic effects of MSCs for change ratio of maximum aortic diameter in AAA models regarding to cell compatibility

Moreover, subgroup analysis regarded to change ratio of maximum aortic diameter were conducted with Spinosa’s [24] study omitted. Results showed allogenic MSCs (SMD = − 2.03, 95% CI [− 2.70, − 1.35]) was more effective than xenogeneic MSCs (SMD = − 1.09, 95% CI [− 1.52, − 0.66]) (*P* = 0.02) (Fig. 7). Moreover, the intraluminal incubation (SMD = − 2.52, 95% CI [− 3.74, − 1.30]) was more efficient than intravenous injection (SMD = − 1.18, 95% CI [− 1.58, − 0.79]) (*P* = 0.04) (Additional file 11: Fig. S9). The results of subgroup analysis regarding to cell source, dose, model induction methods, species, follow-up duration and control type were not statistically significant (Additional file 12: Fig. S10, Additional file 13: Fig. S11 Additional file 14: Fig. S12, Additional file 15: Fig. S13, Additional file 16: Fig. S14, Additional file 17: S15).

### Secondary outcomes

#### Elastin content

A total of 9 studies including 13 experiment arms reported valid data about elastin content of aortic wall after MSCs intervention in AAA models. Random effects model showed MSCs intervention significantly enhanced aortic elastin content compared to control group (SMD = 1.39, 95% CI [0.99, 1.78]) (Additional file 18: Fig. S16).

#### Inflammatory cytokines

To assess the effects of MSCs intervention on expression level of inflammatory cytokines from aortic tissue in AAA animal model, data of MCP-1, IL-6, TNF-α and IL-1β were pooled and analyzed. Eight studies including 12 experimental arms reported MCP-1 expression level, meta-analysis with random effects model showed MCP-1 was significantly suppressed in MSCs group compared to control group (SMD = − 1.78, 95% CI [− 2.81, − 0.76]) (Additional file 19: Fig. S17). A total of 8 studies including 12 experimental arms reported expression level change of TNF-α. Meta-analysis with random effects model showed MSCs intervention could reduce aortic expression level of TNF-α compared with control group (SMD = − 1.23, 95% CI [− 2.091, − 0.37]) (Additional file 20: Fig. S18). Meta analysis of five studies including 8 experimental arms with random effects model showed MSC significantly attenuated IL-6 expression level compared with control group (SMD = − 2.10, 95% CI [− 3.38, − 0.82]) (Additional file 21: Fig. S19). Six studies including 10 experimental arms reported IL-1β level change, however, pooled data with random effects model showed IL-1β expression level was not significantly changed between MSCs group and control group (SMD = − 0.99, 95% CI [− 2.35, 0.38]) (Additional file 22: Fig. S20).

#### Matrix metalloproteinases (MMPs) and tissue inhibitor of metalloproteinases (TIMPs)

We evaluated the effects of MSCs on expression level of MMP2 and MMP9 in AAA models. A total of 5 studies including 9 experimental arms reported data about MMPs levels. Random effects model showed MSCs intervention significantly reduced pro-MMP2 expression level compared to control group (SMD = − 0.93, 95% CI [− 1.44, − 0.42]) (Additional file 23: Fig. S21). Similar results were obtained for active-MMP2 (SMD = − 0.95, 95% CI [− 1.35, − 0.55]), pro-MMP9 (SMD = − 1.00, 95% CI [− 1.35, − 0.64]) and active-MMP9 (SMD = − 1.27, 95% CI [− 1.96, − 0.59]) (Additional file 24: Fig. S22, Additional file 25: Fig. S3, Additional file 26: Fig. S24, respectively,). Moreover, meta analysis of 5 studies including 8 experimental arms with random effects model showed MSC significantly promoted TIMP-1 expression level compared with control group (SMD = 0.48, 95% CI [0.10, 0.86]) (Additional file 27: Fig. S25). Similar results were also obtained for TIMP-2 (SMD = 1.11, 95% CI [0.61, 1.62]) (Additional file 28: Fig. S26).

### Publication bias

**Fig. 8:**
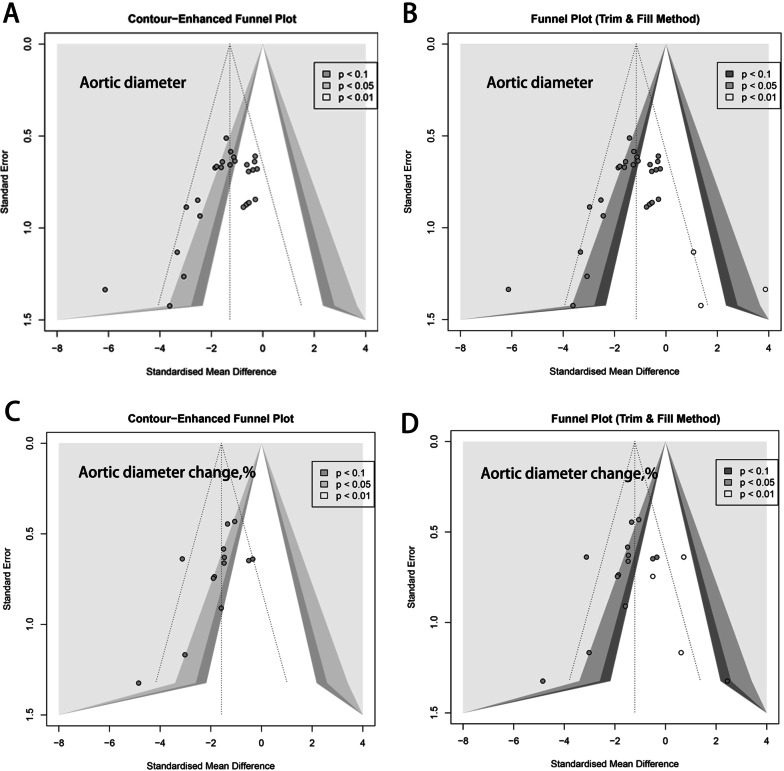
The funnel plots: contour-enhanced funnel plot for **A** maximum aortic diameter and **C** change ratio of maximum aortic diameter, respectively; Trim and fill funnel plot for **B** maximum aortic diameter and **D** change ratio of maximum aortic diameter, respectively

Funnel plots were applied to evaluate publication bias of primary outcomes including maximum aortic diameter and change ratio of maximum aortic diameter. As shown in Fig. 8, the distributions of funnel plots were apparently asymmetric, indicating the presence of potential publication bias. Moreover, trim-and-fill procedures revealed that our initial results were overestimated due to publication bias.

## Discussion

AAA is a progressive and life-threatening pathological process involving complex interactions between vessel cells and molecules, characterized by local modulation of immune reactions, infiltration of inflammatory cells and cytokines, degradation of extracellular matrix, apoptosis and phenotypic transition of VSMCs, etc. [1]. MSCs were known for their abilities of self-renewal, secretion and multipotential differentiation, which empowers them with compacities of modulating immune inflammatory responses, stimulating local cell proliferation and differentiation, and thus promoting tissue regeneration processes [40]. Recent results from preclinical and clinical studies showed MSCs based cell therapy was advantageous in cardiovascular diseases [41]. However, although randomized controlled clinical studies have been registered and implemented to investigate the safety and efficacy of MSC in AAA [42, 43], relevant evidence in AAA is still lacking. To date, plenty preclinical studies have been conducted to investigate the therapeutic potential of MSCs for AAA, given the insufficient number of samples and the large heterogeneity among studies, it is necessary to summarize the present available evidence and further instruct clinical application of MSCs therapy in AAA.

The fundamental mechanisms by which MSCs perform biological repair functions in-vivo mainly including immunomodulatory properties, paracrine effects, homing and differentiation potential [44]. In this meta-analysis, almost all included studies reported the immunomodulatory function of MSC administration in AAA models [15, 16, 21–25, 27, 28, 32, 36], except two studies conducted by the same author [33, 34] who focused on the biomechanical changes of the aortic wall after MSC intervention. Four studies reported paracrine effects of MSC intervention in AAA models, including three studies investigated the conditioned medium of MSC [25, 31, 35] and one study investigated the exosome secreted by MSC [24]. A total of eight studies mentioned MSC capture in aortic tissue after MSC administration using different cell labelling methods [22, 25–30, 36], which indicates MSC migration and homing properties in-vivo. However, only one study by Hosoyama et al. [29] presented definite evidence about MSC locally differentiating into different cell types, which highlights the differentiation abilities of MSC in-vivo. In summary, although the mechanisms of MSC intervention in AAA models have been investigated in different studies, the depth of individual study is not sufficient, subsequent relevant studies should explore the mechanisms in a more systematic and detailed manner.

The progressive increase in aortic diameter is the most apparent and dominant feature of the clinical course in AAA [45]. Notably, our meta-analysis from preclinical studies investigating therapeutic efficacy of MSCs in AAA showed MSCs intervention could suppress aneurysm enlargement and reduce maximum aortic diameter in animals. Elastin is one of the essential extracellular matrix components in aortic wall, fragmentation and degradation of elastin contributes to AAA formation and progression [16], thus maintaining the integrity of arterial elastin is crucial for the prevention of AAA initiation and development [46]. In this present study, meta-analysis showed MSC administration increase the pooled extracellular arterial elastin contents compared to control group. Up-regulation of proinflammatory cytokines and MMPs contributes to aortic immune responses and promotes aortic dilation [47]. In our meta-analysis, the expression level of TNF-α, MCP-1, IL-6, MMP-2 and MMP-9 were suppressed after MSCs intervention in AAA models. Moreover, this study attempted to uncover the different therapeutic efficacy among included studies stratified by detailed study designs, including animal model establishment strategies (animal species, model induction methods) and intervention procedures (cell type, compatibility, route, dose, control type and follow-up duration).

Mesenchymal stem cell has been reported to be isolated from a variety of tissues and body fluids, including bone marrow, adipose tissue, placenta, umbilical cord, blood, urine, etc. [40]. Evidence showed MSCs from different sources exhibited distinct therapeutic effects despite similar bio-characteristics and morphology [48]. In this study, adipose derived MSCs showed better therapeutic efficacy in AAA animals than other cell types regarding to maximum aortic diameter, which may be attributed to the fact that adipose derived MSCs had more potent immunomodulatory effects than other MSCs [49, 50]. Conservatively, given the underlying heterogeneity of the studies included in this study, we believe that more studies are needed to be implemented.

Studies comparing the therapeutic safety and efficacy between allogeneic and xenogeneic MSCs were limited, results of related comparative studies were controversial. María et al. [51] reported that allogeneic and xenogeneic transplantation of adipose derived MSCs shared equal efficacy in acute cerebral infarct model. Fatemeh et al. [52] showed that although there was no statistical difference, allogeneic MSCs transplantation was more efficient than xenogeneic MSCs in the abortion mouse model. One study investigated the immune response to autologous, allogeneic, and xenogeneic MSCs after intra-articular injection in horses, results showed that allogeneic MSCs elicited undetectable immune responses compared with xenogeneic MSCs [53], which highlights the different potential of triggering immune responses between allogeneic and xenogeneic MSCs. In addition, Choi et al. [54] revealed that the strongest humoral immune response was induced by xenogeneic MSCs in a murine systemic lupus erythematosus model compared to allogeneic and syngeneic MSCs. Moreover, Hwang et al. [55] addressed the diverse immunogenic properties and different patterns of immune responses of allogeneic and xenogeneic MSCs in mouse brain. In this study, our results showed significant improvement of allogeneic MSCs compared to xenogeneic MSCs in terms of maximum aortic diameter reduction and diameter change ratio. As mentioned above, the distinct therapeutic efficacy between allogeneic and xenogeneic MSCs in AAA might attribute to the different immunogenic potential since immune responses played the leading role in AAA initiation and progression [56].

Intravenous delivery is the most convenient and the least invasive route, however, the biggest concern for intravenous delivery is the retention in lung and other organs which causes insufficient cell migration and homing [57]. Kanelidis et al. [58] showed that local transendocardial injection of MSCs was superior to intravenous in myocardial infarction. Jeong et al. [59] found intraarterial injection provided increased benefits over local injection and intravenous injection. Apparently, current comparative studies are generally contradictory and insufficient to draw solid conclusion about the optimal delivery route. In our results, perivascular incubation presented the most valid therapeutic efficacy compared to intraluminal and intravenous delivery. The above evidence suggests that the effectiveness of cellular intervention modalities varies in different diseases, and it seems that the closer the first intervention is to the lesion site the more effective it is in AAA, and this observation needs to be corroborated by further studies.

Cell count is an important indicator to assess the clinical efficacy and safety of cell therapy. Kabat et al. [60] summarized MSCs based clinical trials from 2014 to 2018 and found that the minimal effective doses ranging from 70 to 190 million MSCs/patient/dose regarding to intravenous injection. In a meta-analysis investigating the therapeutic efficacy of MSCs in preclinical studies of sepsis, Lalu et al. [61] reported a favorable result of ≥ 1*10^6^ cells dose compared to < 1*10^6^ cells. However, opposite result was achieved in a similar meta-analysis [62]. In our results, it is revealed that ≥ 1*10^6^ cells group was slightly more effective than < 1*10^6^ cells group. Inevitably, current evidence is limited and more investigations should be implemented to get insights into the optimal cell dose of MSC intervention in AAA animals and human studies. Furthermore, during the translational processes from animal to human studies, it is recommended to adjust the optimal dose based on body surface area [63].

In present study, we found the therapeutic efficacy of MSC in AAA was decreasing over time duration, the most significant diameter decrease was achieved in ≤ 2 week (SMD = − 1.47) group, and it was more inferior in ≤ 4 weeks group (SMD = − 1.20), the result of > 4 weeks group was not significant. It is undeniable that all available animal models have limitations regarding to the pathological interpretation and natural history of human AAA [58], the subsequent time-dependent self-healing process exits in most AAA animal models once the stimulus stops [64]. However, their translational relevance should not be doubted, and above results underscore the necessity of developing novel AAA animal models and highlight the investigation of optimal intervention frequency in both animal and human AAA studies.

Although the results of our study are encouraging and instructive, several definite limitations exist. First, funnel plots revealed publication bias in this study, which indicates some missing data in the whole study and more relevant studies are needed. Second, the heterogeneity among studies was significant and individual studies was omitted to achieve eligible between-study heterogeneity, which might affect the accuracy of this study. Moreover, the quality assessments of included studies are relatively low since most studies lacked blinding and randomization methods. Furthermore, our meta-analysis focused on the diameter change as primary outcome, while most studies provided values of the final maximum aortic diameter, some studies reported the percentage change of maximum aortic diameter, which may contribute to the instability of our results. In addition, for secondary outcomes evaluation, too few studies provided available data about elastin contents and inflammatory cytokines, thus, it is suggested to be cautious when interpreting the results. Finally, considering the potential heterogeneity in animal studies, the random effects model was applied throughout this meta-analysis, which might overestimate the overall effects of MSC in AAA.

## Conclusion

The present meta-analysis and systematic review included 18 studies, results showed that MSCs intervention is associated with suppression of aortic diameter enlargement, reduction of elastin degradation and inflammatory cytokines such as MCP-1, IL-6, TNF-α and MMP2/9, increase of TIMP1/2 expression. Regarding this, future large-scale animal studies are needed to determine the eligible protocols of transplantation details. Furthermore, clinical randomized investigations are required to improve current insight on the therapeutic efficacy of MSCs in AAA patients, thereby, potentially promoting clinical application of MSC based cell therapy in AAA.

## Supplementary Information

**Additional file 1**. PRISMA checklist.

**Additional file 2**. Search strategy.

**Additional file 3: Fig. S1**. Forest plot summarizing the relationship between MSCs intervention route and diameter in preclinical models of AAA.

**Additional file 4: Fig. S2**. Forest plot summarizing the relationship between MSCs cell dose and diameter in preclinical models of AAA.

**Additional file 5: Fig. S3**. Forest plot summarizing the relationship between follow-up duration and diameter in preclinical models of AAA.

**Additional file 6: Fig. S4**. Forest plot summarizing the relationship between model induction methods and diameter in preclinical models of AAA.

**Additional file 7: Fig. S5**. Forest plot summarizing the relationship between animal species and diameter in preclinical models of AAA.

**Additional file 8: Fig. S6**. Forest plot summarizing the relationship between control type and diameter in preclinical models of AAA.

**Additional file 9: Fig. S7**. The original forest plot of the therapeutic effects of MSCs for maximum aortic diameter change ratio (% increase) in AAA models, compared with control group.

**Additional file 10: Fig. S8**. Identification of outlier study regarding to maximum aortic diameter change ratio (% increase). **A**: Baujat plot. **B**: Influence diagnostics. **C, D**: Leave-one-out meta-analysis ranked by effect size and I2, respectively. **E, F**: GOSH and GOSH diagnostic (k-means algorithm) plots, respectively.

**Additional file 11: Fig. S9**. Forest plot summarizing the relationship between MSCs intervention route and diameter change ratio (% increase) in preclinical models of AAA.

**Additional file 12: Fig. S10**. Forest plot summarizing the relationship between MSCs cell source and diameter change ratio (% increase) in preclinical models of AAA.

**Additional file 13: Fig. S11**. Forest plot summarizing the relationship between MSCs cell dose and diameter change ratio (% increase) in preclinical models of AAA.

**Additional file 14: Fig. S12**. Forest plot summarizing the relationship between model induction methods and diameter change ratio (% increase) in preclinical models of AAA.

**Additional file 15: Fig. S13**. Forest plot summarizing the relationship between animal species and diameter change ratio (% increase) in preclinical models of AAA.

**Additional file 16: Fig. S14**. Forest plot summarizing the relationship between follow-up duration and diameter change ratio (% increase) in preclinical models of AAA.

**Additional file 17: Fig. S15**. Forest plot summarizing the relationship between control type and diameter change ratio (% increase) in preclinical models of AAA.

**Additional file 18: Fig. S16**. Forest plot of the therapeutic effects of MSCs for elastin content in AAA models, compared with control group.

**Additional file 19: Fig. S17**. Forest plot of the therapeutic effects of MSCs for MCP-1 level in AAA models, compared with control group.

**Additional file 20: Fig. S18**. Forest plot of the therapeutic effects of MSCs for TNF-α level in AAA models, compared with control group.

**Additional file 21: Fig. S19**. Forest plot of the therapeutic effects of MSCs for IL-6 level in AAA models, compared with control group.

**Additional file 22: Fig. S20**. Forest plot of the therapeutic effects of MSCs for IL-1β level in AAA models, compared with control group.

**Additional file 23: Fig. S21**. Forest plot of the therapeutic effects of MSCs for pro-MMP2 level in AAA models, compared with control group.

**Additional file 24: Fig. S22**. Forest plot of the therapeutic effects of MSCs for active-MMP2 level in AAA models, compared with control group.

**Additional file 25: Fig. S23**. Forest plot of the therapeutic effects of MSCs for pro-MMP9 level in AAA models, compared with control group.

**Additional file 26: Fig. S24**. Forest plot of the therapeutic effects of MSCs for active-MMP9 level in AAA models, compared with control group.

**Additional file 27: Fig. S25**. Forest plot of the therapeutic effects of MSCs for TIMP-1 level in AAA models, compared with control group.

**Additional file 28: Fig. S26**. Forest plot of the therapeutic effects of MSCs for TIMP-2 level in AAA models, compared with control group.

## Data Availability

The included studies and data were publicly available and can be accessed from the publishers.

## Abbreviations

AAA: Abdominal aortic aneurysm
MSC: Mesenchymal stem cell
VSMC: Vascular smooth muscle cell
AngII: Angiotensin II
apoE−/−: Apolipoprotein E knockout mice
PBS: Phosphate buffered saline
NS: Not mentioned
MCP-1: Monocyte chemoattractant protein-1
TNF-α: Tumor necrosis factor-α
IL-1β: Interleukin-1β
IL-6: Interleukin-6
MMP-2: Matrix metalloproteinase 2
MMP-9: Matrix metalloproteinase 9
TIMP-1: Tissue inhibitor of metalloproteinase-1
TIMP-2: Tissue inhibitor of metalloproteinase-2
PRISMA: Preferred Reporting Items for Systematic Reviews and Meta-analyses
SYRCLE: Systematic Review Centre for Laboratory Animal Experimentation
SMD: Standardized mean difference
