# Supplementary material for: Therapeutic efficacy of mesenchymal stem cells for abdominal aortic aneurysm: a meta-analysis of preclinical studies
Source: Stem Cell Res Ther. 2022 Feb 24;13:81. doi: 10.1186/s13287-022-02755-w (PMC8867868; doi:10.1186/s13287-022-02755-w)
Supplement: Supplementary file 2 — Additional file 2. Search strategy. [file 13287_2022_2755_MOESM2_ESM.docx]

**Pubmed**

| **ID** | **Search** | **Hits** |
| --- | --- | --- |
| #1 | Aortic Aneurysm, Abdominal[MeSH Terms] | 20,552 |
| #2 | (((((Aortic Aneurysm, Abdominal[Title/Abstract]) OR (Abdominal Aortic Aneurysms[Title/Abstract])) OR (Aneurysms, Abdominal Aortic[Title/Abstract])) OR (Aortic Aneurysms, Abdominal[Title/Abstract])) OR (Abdominal Aortic Aneurysm[Title/Abstract])) OR (Aneurysm, Abdominal Aortic[Title/Abstract]) | 19,717 |
| #3 | #1 OR #2 | 26,342 |
| #4 | ((((Stem Cells[MeSH Terms]) OR (Stromal Cells[MeSH Terms])) ) OR (Stem Cell Research[MeSH Terms])) OR (Cell Transplantation[MeSH Terms]) | 329,474 |
| #5 | (((((((((((((((((((((((((((Stem Cells[Title/Abstract]) OR (Cell, Stem[Title/Abstract])) OR (Cells, Stem[Title/Abstract])) OR (Stem Cell[Title/Abstract])) OR (Progenitor Cells[Title/Abstract])) OR (Cell, Progenitor[Title/Abstract])) OR (Cells, Progenitor[Title/Abstract])) OR (Progenitor Cell[Title/Abstract])) OR (Mother Cells[Title/Abstract])) OR (Cell, Mother[Title/Abstract])) OR (Cells, Mother[Title/Abstract])) OR (Mother Cell[Title/Abstract])) OR (Colony-Forming Unit[Title/Abstract])) OR (Colony Forming Unit[Title/Abstract])) OR (Colony-Forming Units[Title/Abstract])) OR (Colony Forming Units[Title/Abstract])) OR (Cell Transplantation[Title/Abstract])) OR (Transplantation, Cell[Title/Abstract])) OR (Stem Cell Research[Title/Abstract])) OR (Research, Stem Cell[Title/Abstract])) OR (Researchs, Stem Cell[Title/Abstract])) OR (Stem Cell Researchs[Title/Abstract])) OR (Embryonic Stem Cell Research[Title/Abstract])) OR (Adult Stem Cell Research[Title/Abstract])) OR (Stromal Cells[Title/Abstract])) OR (Cell, Stromal[Title/Abstract])) OR (Cells, Stromal[Title/Abstract])) OR (Stromal Cell[Title/Abstract]) | 403,594 |
| #6 | ((((stem cell*[Title/Abstract]) OR (stromal cell*[Title/Abstract])) OR (progenitor cell*[Title/Abstract])) OR (precusor cell*[Title/Abstract])) OR (cell* therap*[Title/Abstract]) | 386,299 |
| #7 | #4 OR #5 OR #6 | 505,906 |
| #8 | #3 AND #7 | 84 |
| **Last Run Date: 25/10/2021** | | |

**EBSCO**

| **ID** | **Search** | **Hits** |
| --- | --- | --- |
| #1 | TI ( Aortic Aneurysm, Abdominal OR Abdominal Aortic Aneurysms OR Aneurysms, Abdominal Aortic OR Aortic Aneurysms, Abdominal OR Abdominal Aortic Aneurysm OR Aneurysm, Abdominal Aortic ) OR AB ( Aortic Aneurysm, Abdominal OR Abdominal Aortic Aneurysms OR Aneurysms, Abdominal Aortic OR Aortic Aneurysms, Abdominal OR Abdominal Aortic Aneurysm OR Aneurysm, Abdominal Aortic ) | 1468 |
| #2 | TI ( Stem Cells OR Cell, Stem OR Cells, Stem OR Stem Cell OR Progenitor Cells OR Cell, Progenitor OR Cells, Progenitor OR Progenitor Cell OR Mother Cells OR Cell, Mother OR Cells, Mother OR Mother Cell OR Colony-Forming Unit OR Colony Forming Unit OR Colony-Forming Units OR Colony Forming Units OR Cell Transplantation OR Transplantation, Cell OR Stem Cell Research OR Research, Stem Cell OR Researchs, Stem Cell OR Stem Cell Researchs OR Embryonic Stem Cell Research OR Adult Stem Cell Research OR Stromal Cells OR Cell, Stromal OR Cells, Stromal OR Stromal Cell ) OR AB ( Stem Cells OR Cell, Stem OR Cells, Stem OR Stem Cell OR Progenitor Cells OR Cell, Progenitor OR Cells, Progenitor OR Progenitor Cell OR Mother Cells OR Cell, Mother OR Cells, Mother OR Mother Cell OR Colony-Forming Unit OR Colony Forming Unit OR Colony-Forming Units OR Colony Forming Units OR Cell Transplantation OR Transplantation, Cell OR Stem Cell Research OR Research, Stem Cell OR Researchs, Stem Cell OR Stem Cell Researchs OR Embryonic Stem Cell Research OR Adult Stem Cell Research OR Stromal Cells OR Cell, Stromal OR Cells, Stromal OR Stromal Cell ) | 49260 |
| #3 | TI ( stem cell* OR stromal cell* OR progenitor cell* OR precusor cell* OR cell* therap* ) OR AB ( stem cell* OR stromal cell* OR progenitor cell* OR precusor cell* OR cell* therap* ) | 83894 |
| #4 | #2 OR #3 | 50850 |
| #5 | #1 AND #4 | 6 |
| **Last Run Date: 25/10/2021** | | |

**EMBASE**

| **ID** | **Search** | **Hits** |
| --- | --- | --- |
| #1 | 'abdominal aortic aneurysm'/exp | 31501 |
| #2 | 'aortic aneurysm, abdominal':ti,ab,kw OR 'abdominal aortic aneurysms':ti,ab,kw OR 'aneurysms, abdominal aortic':ti,ab,kw OR 'aortic aneurysms, abdominal':ti,ab,kw OR 'abdominal aortic aneurysm':ti,ab,kw OR 'aneurysm, abdominal aortic':ti,ab,kw | 25635 |
| #3 | #1 OR #2 | 35264 |
| #4 | 'stem cell'/exp | 414427 |
| #5 | 'stem cells':ti,ab,kw OR 'cells, stem':ti,ab,kw OR 'stem cell':ti,ab,kw OR 'progenitor cells':ti,ab,kw OR 'cell, progenitor':ti,ab,kw OR 'cells, progenitor':ti,ab,kw OR 'progenitor cell':ti,ab,kw OR 'mother cells':ti,ab,kw OR 'cell, mother':ti,ab,kw OR 'cells, mother':ti,ab,kw OR 'mother cell':ti,ab,kw OR 'colony-forming unit':ti,ab,kw OR 'colony forming unit':ti,ab,kw OR 'colony-forming units':ti,ab,kw OR 'colony forming units':ti,ab,kw | 526653 |
| #6 | #4 OR #5 | 612439 |
| #7 | 'cell transplantation'/exp | 190800 |
| #8 | 'cell transplantation':ti,ab,kw OR 'transplantation, cell':ti,ab,kw | 109168 |
| #9 | #7 OR #8 | 203941 |
| #10 | 'stem cell research'/exp | 2558 |
| #11 | 'stem cell research':ti,ab,kw OR 'research, stem cell':ti,ab,kw OR 'researchs, stem cell':ti,ab,kw OR 'stem cell researchs':ti,ab,kw OR 'embryonic stem cell research':ti,ab,kw OR 'adult stem cell research':ti,ab,kw | 4513 |
| #12 | 'stroma cell'/exp | 56668 |
| #13 | 'stromal cells':ti,ab,kw OR 'cell, stromal':ti,ab,kw OR 'cells, stromal':ti,ab,kw OR 'stromal cell':ti,ab,kw | 64160 |
| #14 | #10 OR #11 | 5883 |
| #15 | #12 OR #13 | 79879 |
| #16 | 'stem cell*':ti,ab,kw OR 'stromal cell*':ti,ab,kw OR 'progenitor cell*':ti,ab,kw OR 'precusor cell*':ti,ab,kw OR 'cell* therap*':ti,ab,kw | 573895 |
| #17 | #6 OR #9 OR #14 OR #15 OR #16 | 739600 |
| #18 | #3 AND #17 | 207 |
| **Last Run Date: 25/10/2021** | | |

**Web of Science**

| **ID** | **Search** | **Hits** |
| --- | --- | --- |
| #1 | TS=(Aortic Aneurysm, Abdominal OR Abdominal Aortic Aneurysms OR Aneurysms, Abdominal Aortic OR Aortic Aneurysms, Abdominal OR Abdominal Aortic Aneurysm OR Aneurysm, Abdominal Aortic) | 20598 |
| #2 | TS=(Stem Cells OR Cell, Stem OR Cells, Stem OR Stem Cell OR Progenitor Cells OR Cell, Progenitor OR Cells, Progenitor OR Progenitor Cell OR Mother Cells OR Cell, Mother OR Cells, Mother OR Mother Cell OR Colony-Forming Unit OR Colony Forming Unit OR Colony-Forming Units OR Colony Forming Units OR Cell Transplantation OR Transplantation, Cell OR Stem Cell Research OR Research, Stem Cell OR Researchs, Stem Cell OR Stem Cell Researchs OR Embryonic Stem Cell Research OR Adult Stem Cell Research OR Stromal Cells OR Cell, Stromal OR Cells, Stromal OR Stromal Cell) | 683959 |
| #3 | TS=(stem cell* OR stromal cell* OR progenitor cell* OR precusor cell* OR cell* therap*) | 1392110 |
| #4 | #3 OR #2 | 1467347 |
| #5 | #4 AND #1 | 888 |
| **Last Run Date: 25/10/2021** | | |

**Cochrane**

| **ID** | **Search** | **Hits** |
| --- | --- | --- |
| #1 | MeSH descriptor: [Aortic Aneurysm, Abdominal] explode all trees | 597 |
| #2 | Aortic Aneurysm, Abdominal OR Abdominal Aortic Aneurysms OR Aneurysms, Abdominal Aortic OR Aortic Aneurysms, Abdominal OR Abdominal Aortic Aneurysm OR Aneurysm, Abdominal Aortic | 1483 |
| #3 | #1 OR #2 | 1483 |
| #4 | MeSH descriptor: [Stem Cells] explode all trees | 846 |
| #5 | MeSH descriptor: [Cell Transplantation] explode all trees | 2214 |
| #6 | MeSH descriptor: [Stromal Cells] explode all trees | 192 |
| #7 | MeSH descriptor: [Stem Cell Research] explode all trees | 0 |
| #8 | Stem Cells OR Cell, Stem OR Cells, Stem OR Stem Cell OR Progenitor Cells OR Cell, Progenitor OR Cells, Progenitor OR Progenitor Cell OR Mother Cells OR Cell, Mother OR Cells, Mother OR Mother Cell OR Colony-Forming Unit OR Colony Forming Unit OR Colony-Forming Units OR Colony Forming Units OR Cell Transplantation OR Transplantation, Cell OR Stem Cell Research OR Research, Stem Cell OR Researchs, Stem Cell OR Stem Cell Researchs OR Embryonic Stem Cell Research OR Adult Stem Cell Research OR Stromal Cells OR Cell, Stromal OR Cells, Stromal OR Stromal Cell | 23741 |
| #9 | stem cell* OR stromal cell* OR progenitor cell* OR precusor cell* OR cell* therap* | 104280 |
| #10 | #4 OR #5 OR #6 OR #7 OR #8 OR #9 | 107923 |
| #11 | #3 AND #10 | 93 |
| **Last Run Date: 25/10/2021** | | |
