# Supplementary figures and images for: Therapeutic efficacy of mesenchymal stem cells for abdominal aortic aneurysm: a meta-analysis of preclinical studies
Source: Stem Cell Res Ther. 2022 Feb 24;13:81. doi: 10.1186/s13287-022-02755-w (PMC8867868; doi:10.1186/s13287-022-02755-w)

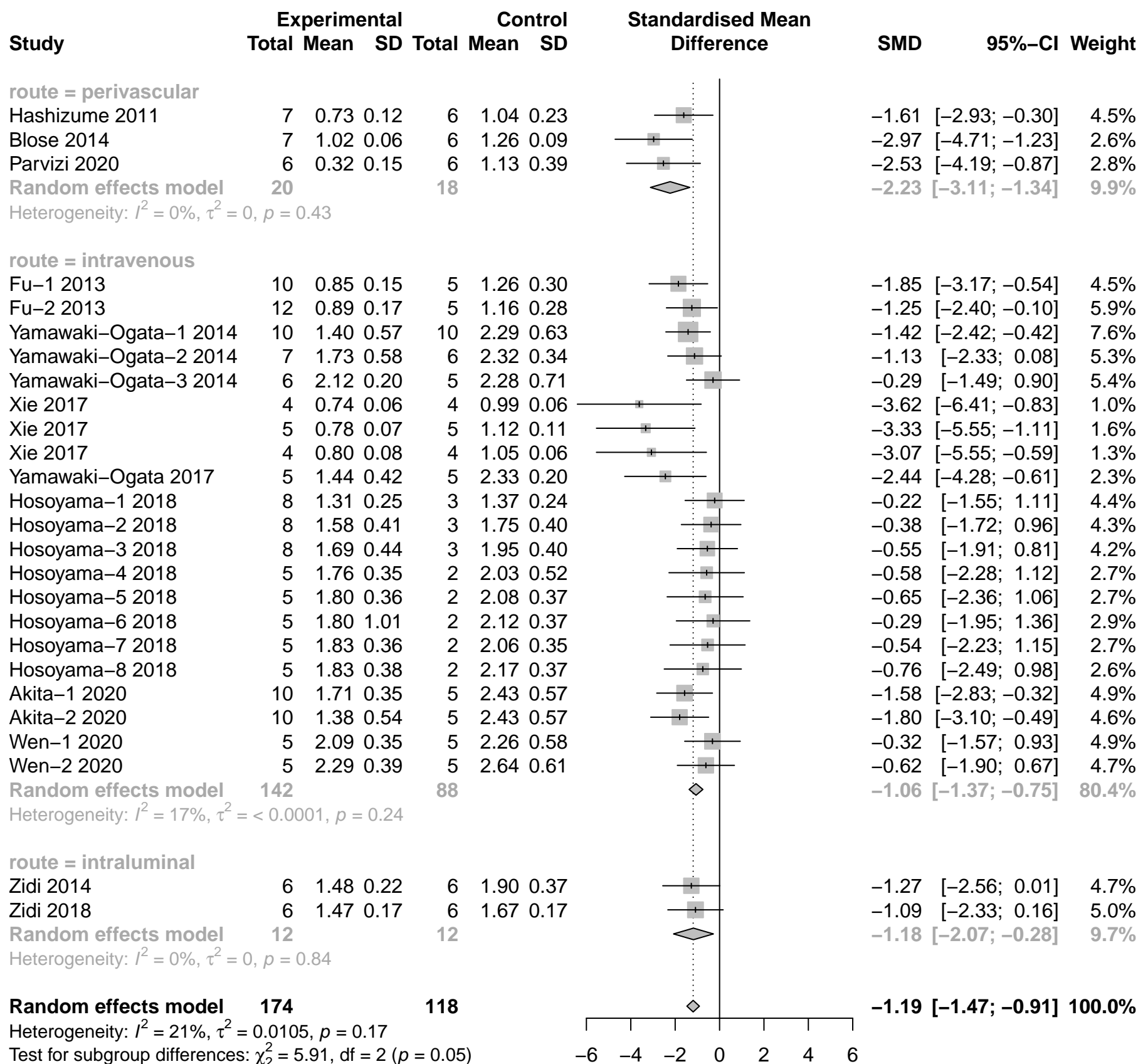

Supplement: Supplementary file 3 — Additional file 3: Fig. S1. Forest plot summarizing the relationship between MSCs intervention route and diameter in preclinical models of AAA. [file 13287_2022_2755_MOESM3_ESM.pdf]

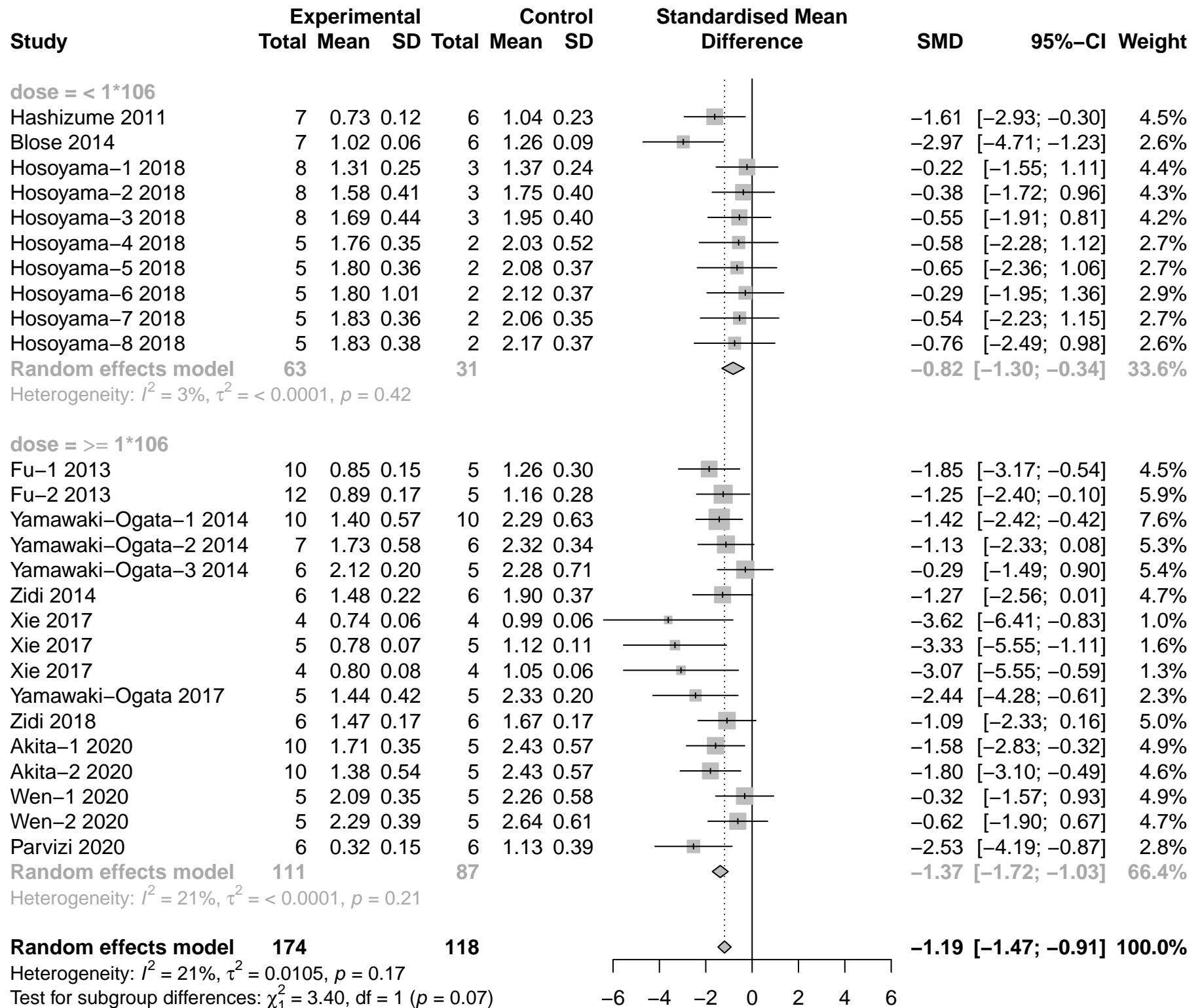

Supplement: Supplementary file 4 — Additional file 4: Fig. S2. Forest plot summarizing the relationship between MSCs cell dose and diameter in preclinical models of AAA. [file 13287_2022_2755_MOESM4_ESM.pdf]

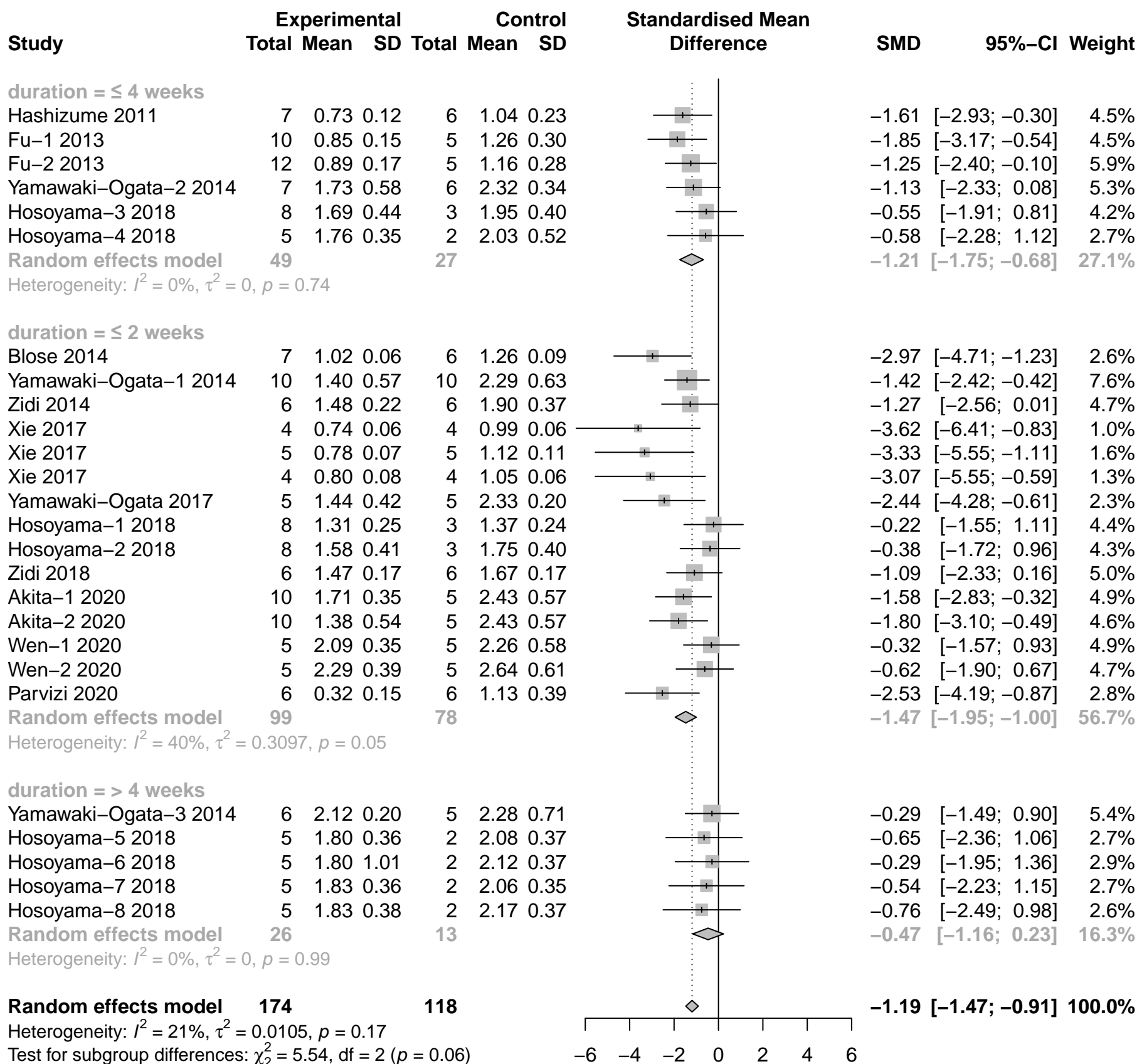

Supplement: Supplementary file 5 — Additional file 5: Fig. S3. Forest plot summarizing the relationship between follow-up duration and diameter in preclinical models of AAA. [file 13287_2022_2755_MOESM5_ESM.pdf]

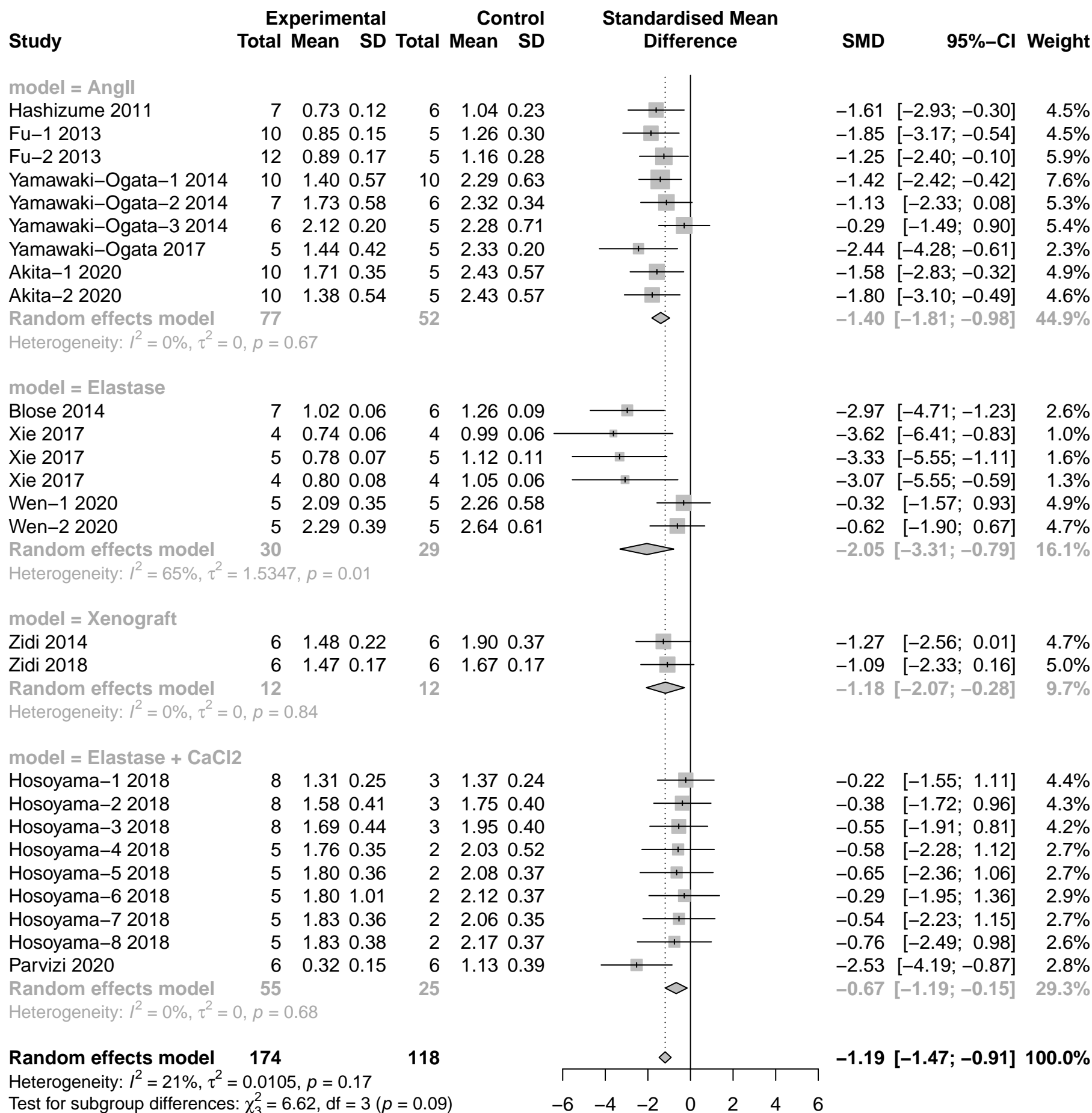

Supplement: Supplementary file 6 — Additional file 6: Fig. S4. Forest plot summarizing the relationship between model induction methods and diameter in preclinical models of AAA. [file 13287_2022_2755_MOESM6_ESM.pdf]

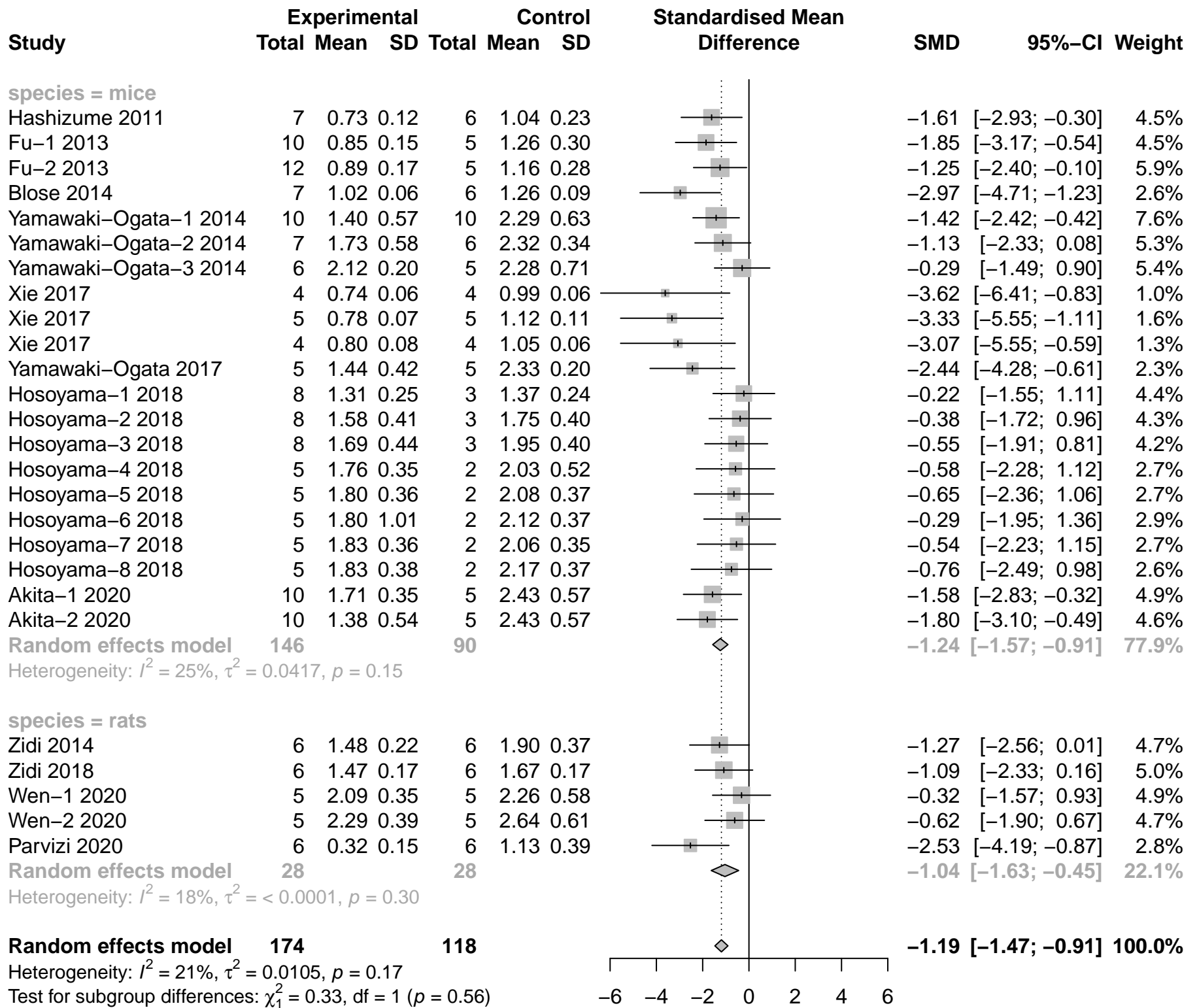

Supplement: Supplementary file 7 — Additional file 7: Fig. S5. Forest plot summarizing the relationship between animal species and diameter in preclinical models of AAA. [file 13287_2022_2755_MOESM7_ESM.pdf]

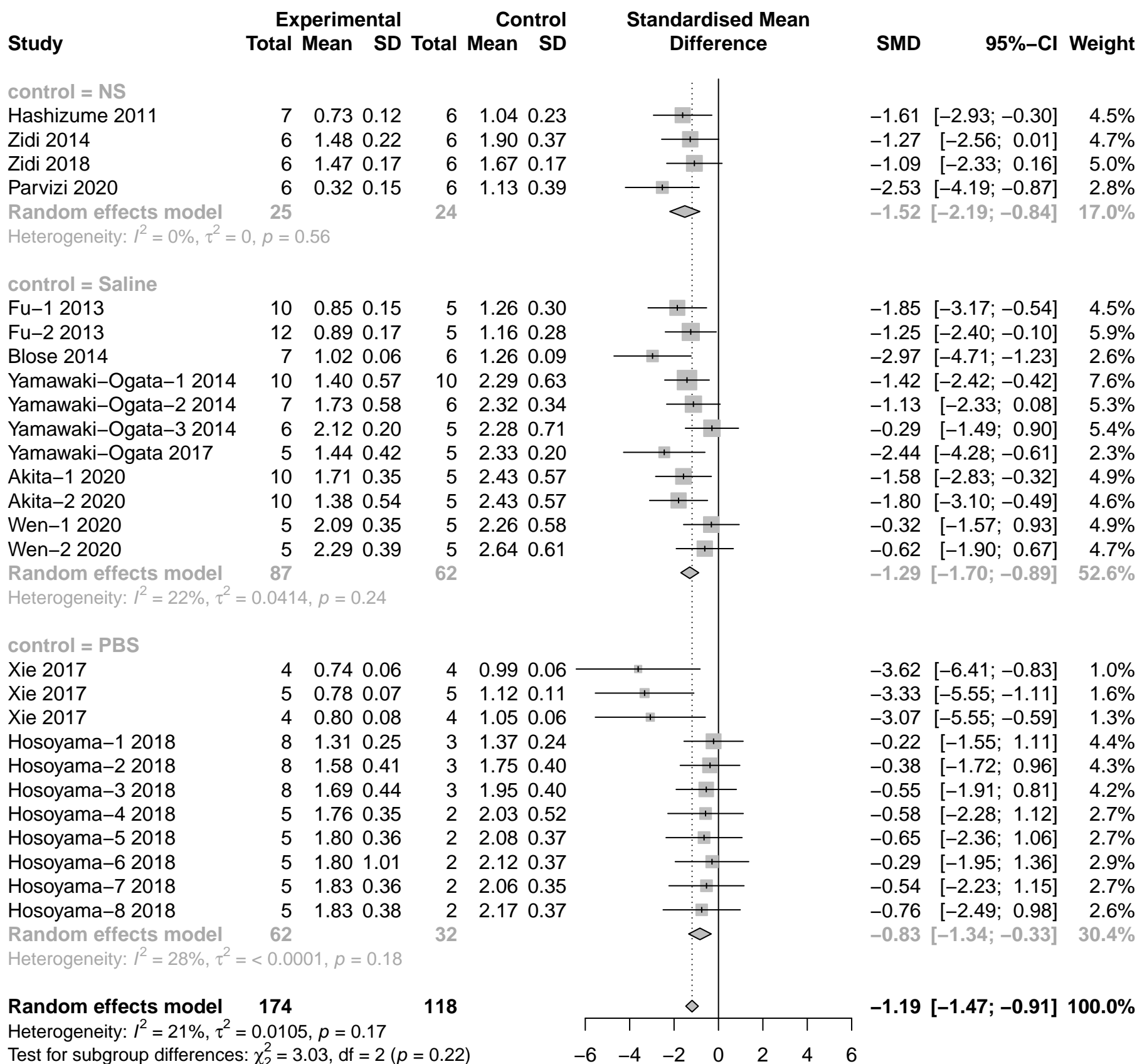

Supplement: Supplementary file 8 — Additional file 8: Fig. S6. Forest plot summarizing the relationship between control type and diameter in preclinical models of AAA. [file 13287_2022_2755_MOESM8_ESM.pdf]

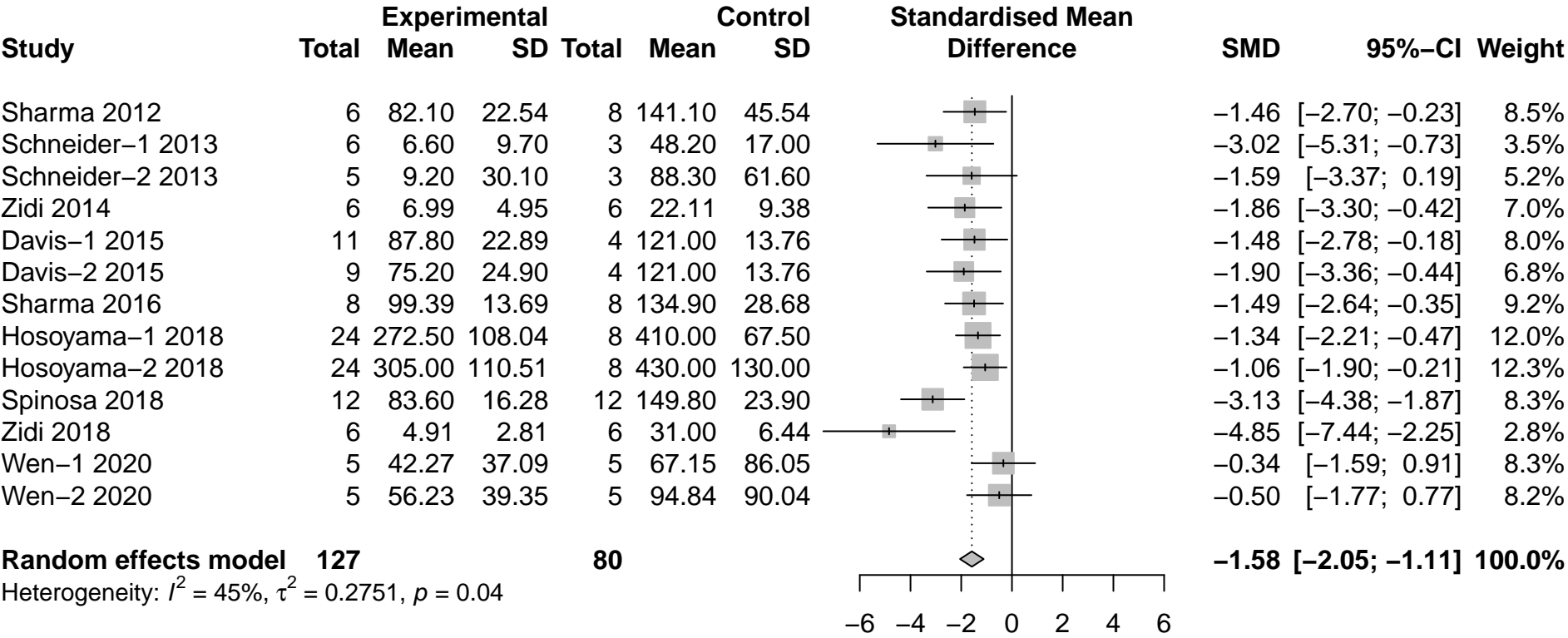

Supplement: Supplementary file 9 — Additional file 9: Fig. S7. The original forest plot of the therapeutic effects of MSCs for maximum aortic diameter change ratio (% increase) in AAA models, compared with control group. [file 13287_2022_2755_MOESM9_ESM.pdf]

A

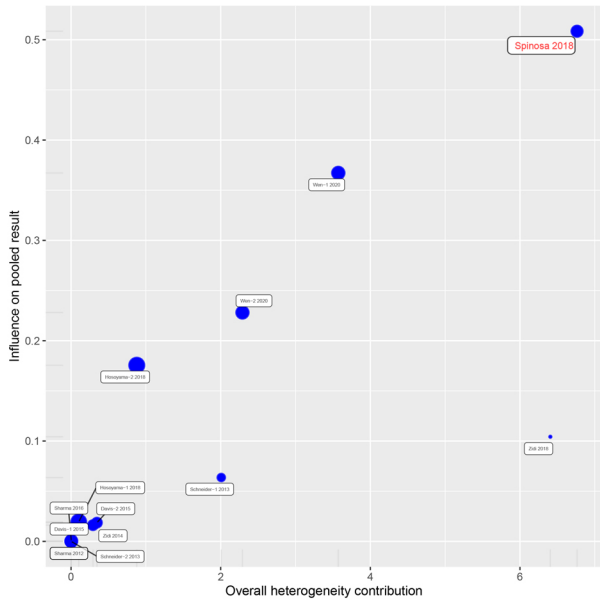

B

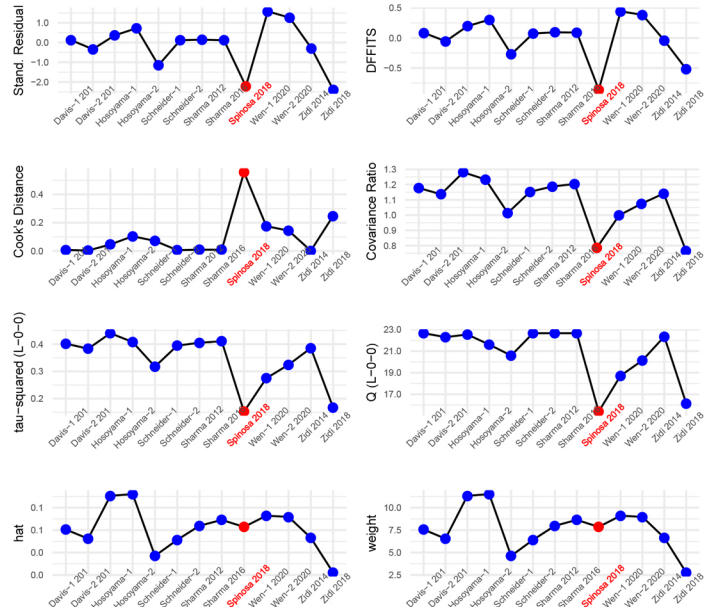

C

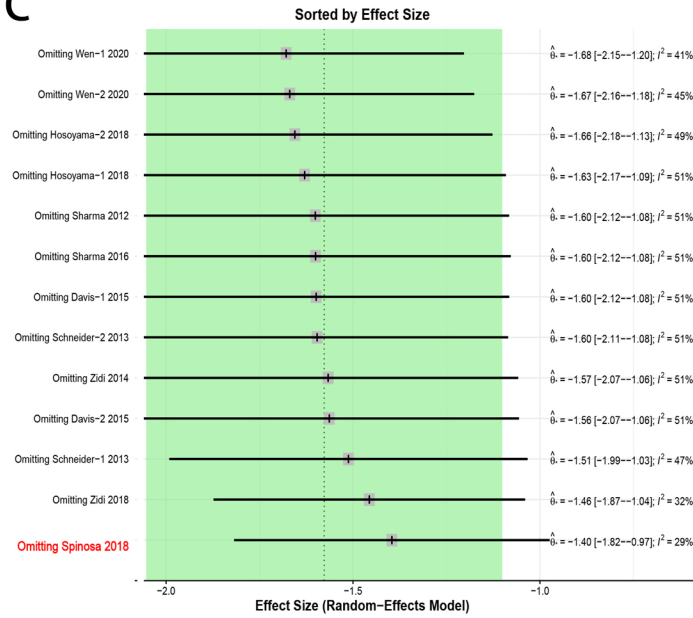

D

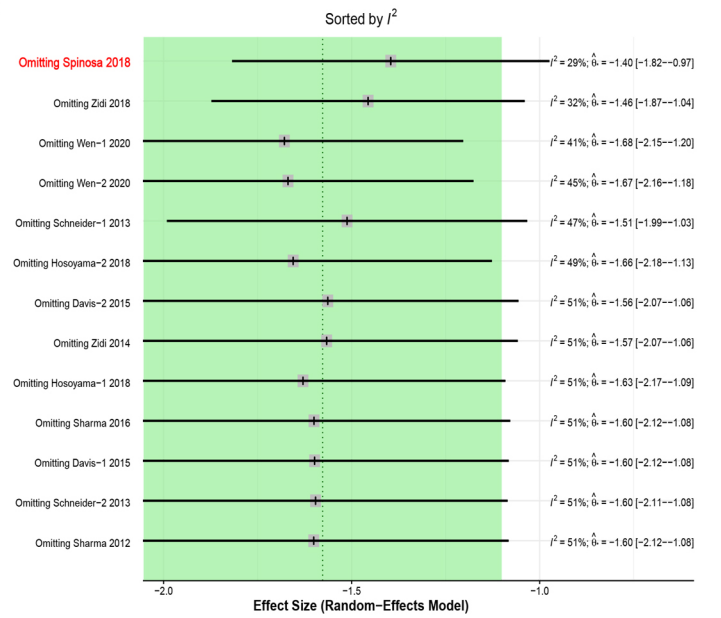

E

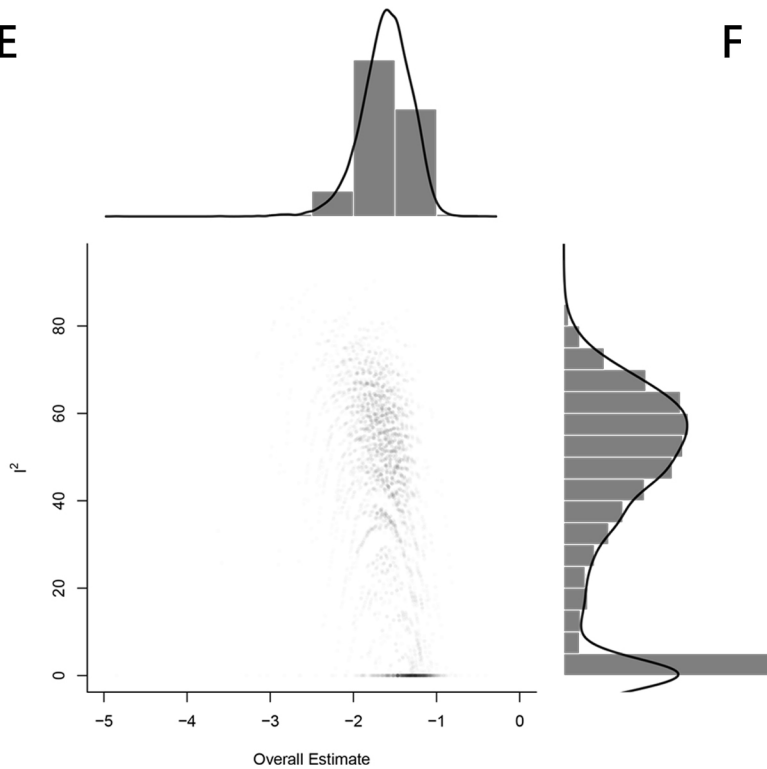

F

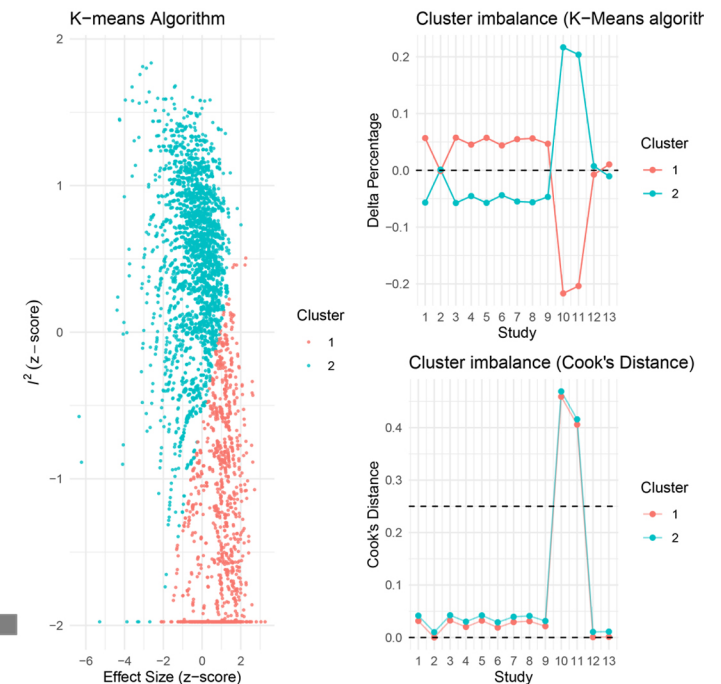

Supplement: Supplementary file 10 — Additional file 10: Fig. S8. Identification of outlier study regarding to maximum aortic diameter change ratio (% increase). A: Baujat plot. B: Influence diagnostics. C, D: Leave-one-out meta-analysis ranked by effect size and I2, respectively. E, F: GOSH and GOSH diagnostic (k-means algorithm) plots, respectively. [file 13287_2022_2755_MOESM10_ESM.pdf]

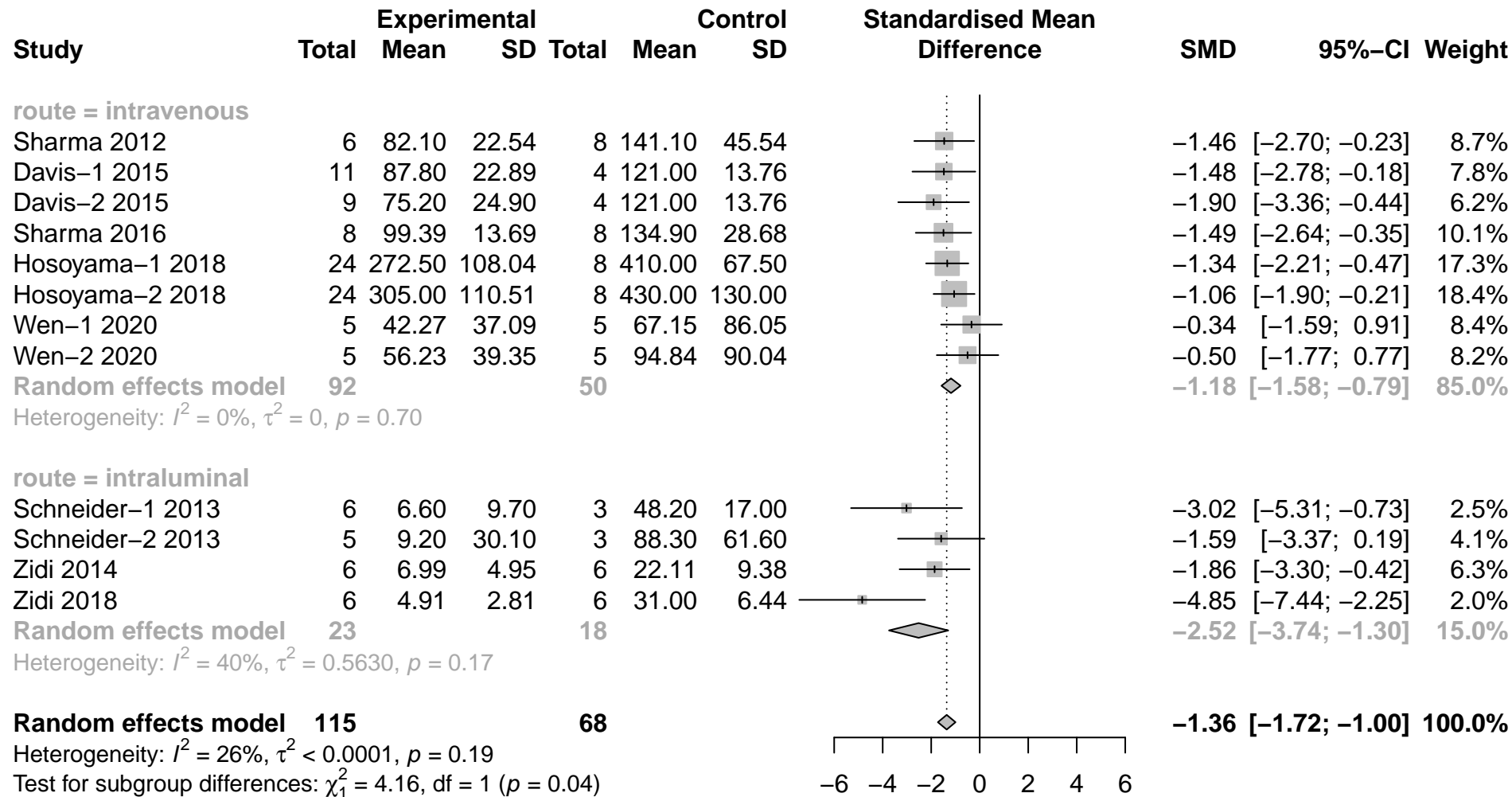

Supplement: Supplementary file 11 — Additional file 11: Fig. S9. Forest plot summarizing the relationship between MSCs intervention route and diameter change ratio (% increase) in preclinical models of AAA. [file 13287_2022_2755_MOESM11_ESM.pdf]

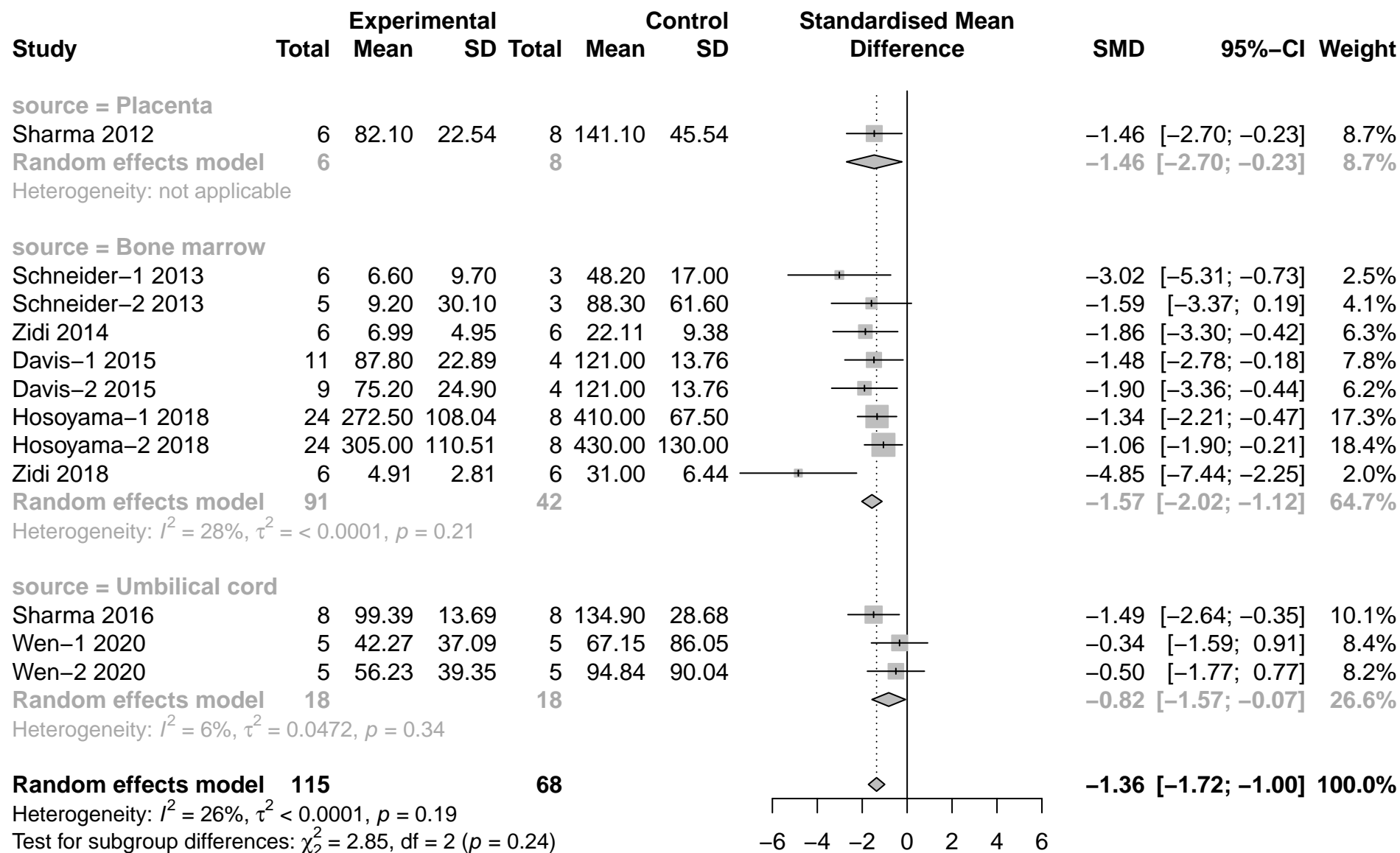

Supplement: Supplementary file 12 — Additional file 12: Fig. S10. Forest plot summarizing the relationship between MSCs cell source and diameter change ratio (% increase) in preclinical models of AAA. [file 13287_2022_2755_MOESM12_ESM.pdf]

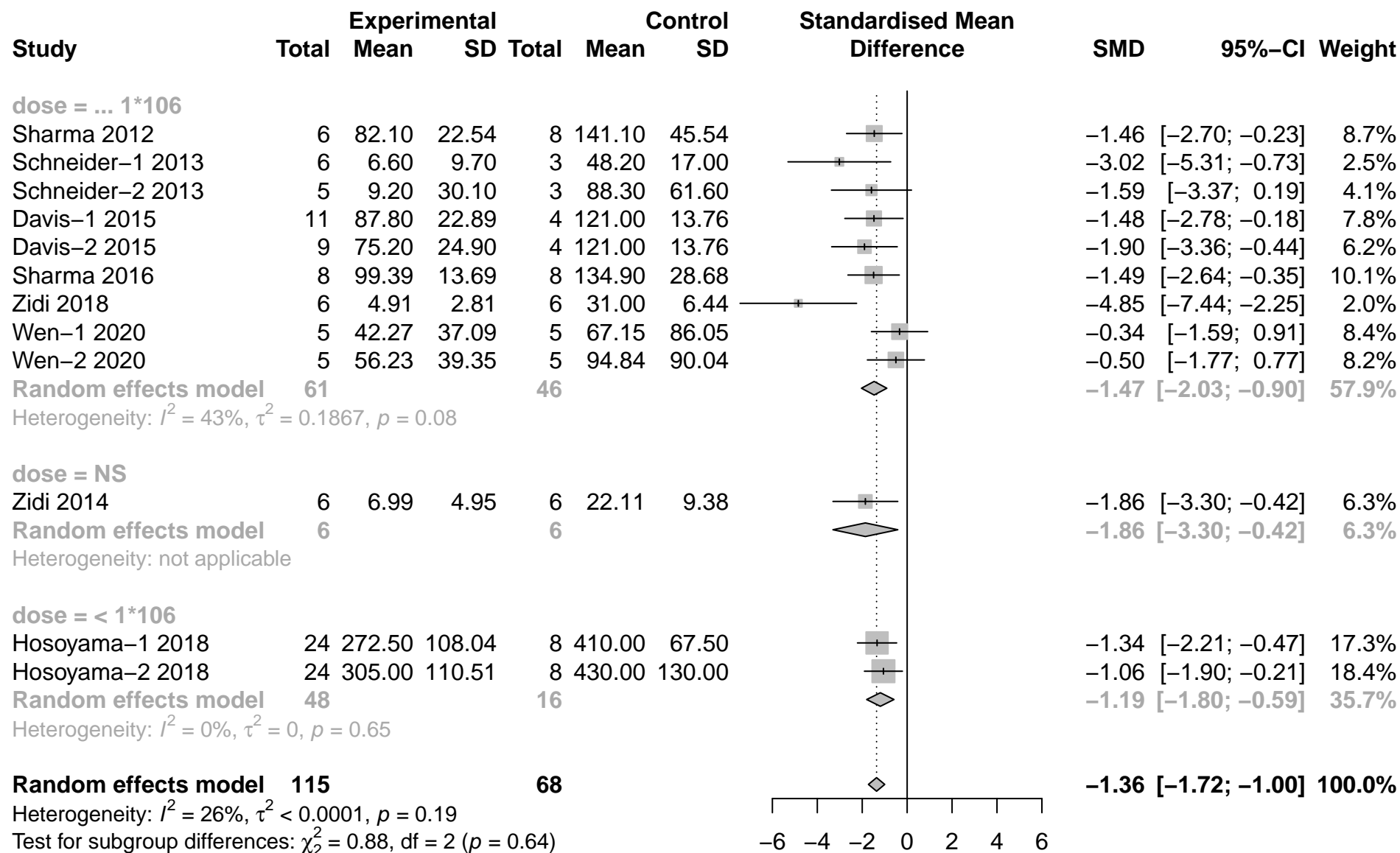

Supplement: Supplementary file 13 — Additional file 13: Fig. S11. Forest plot summarizing the relationship between MSCs cell dose and diameter change ratio (% increase) in preclinical models of AAA. [file 13287_2022_2755_MOESM13_ESM.pdf]

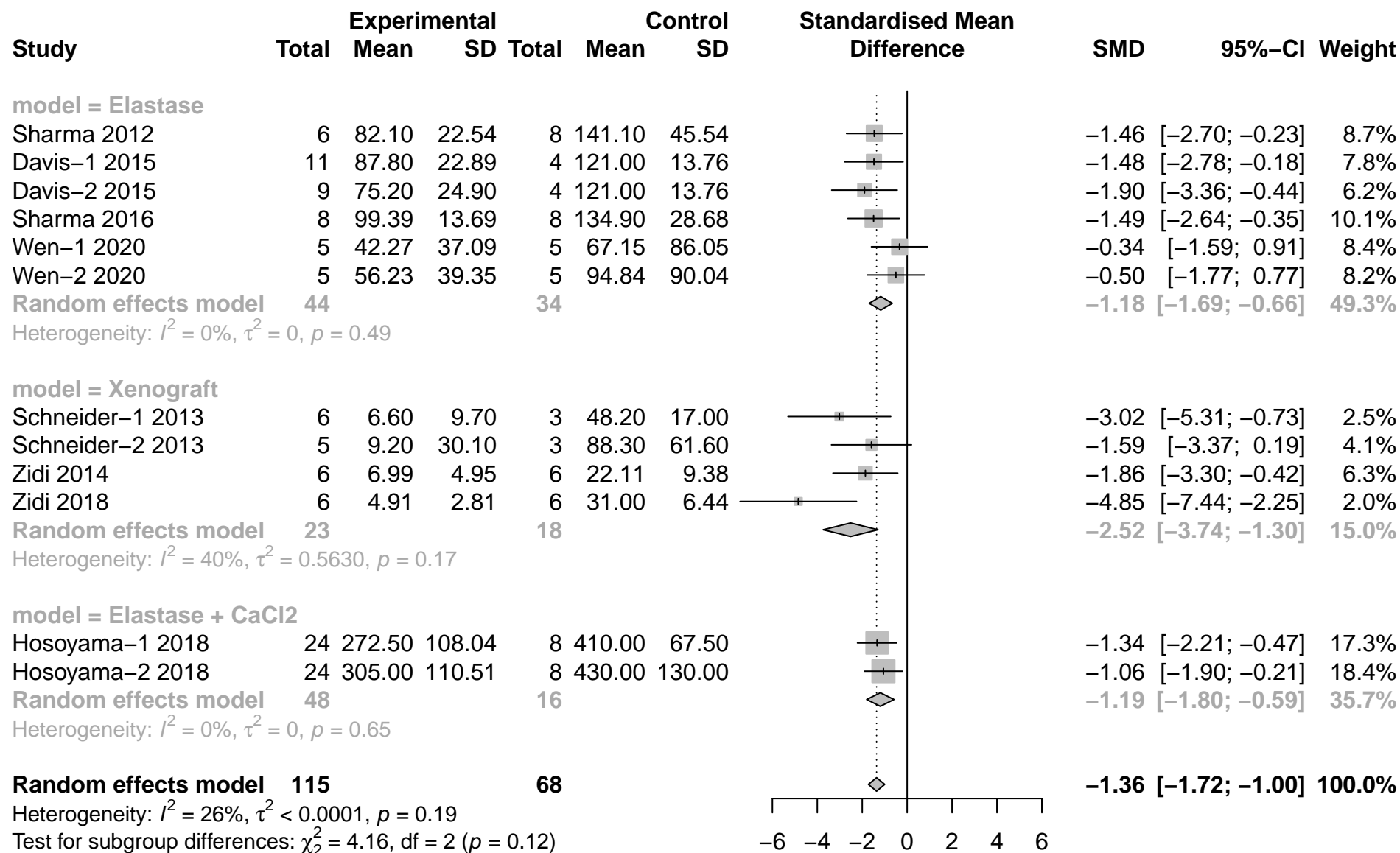

Supplement: Supplementary file 14 — Additional file 14: Fig. S12. Forest plot summarizing the relationship between model induction methods and diameter change ratio (% increase) in preclinical models of AAA. [file 13287_2022_2755_MOESM14_ESM.pdf]

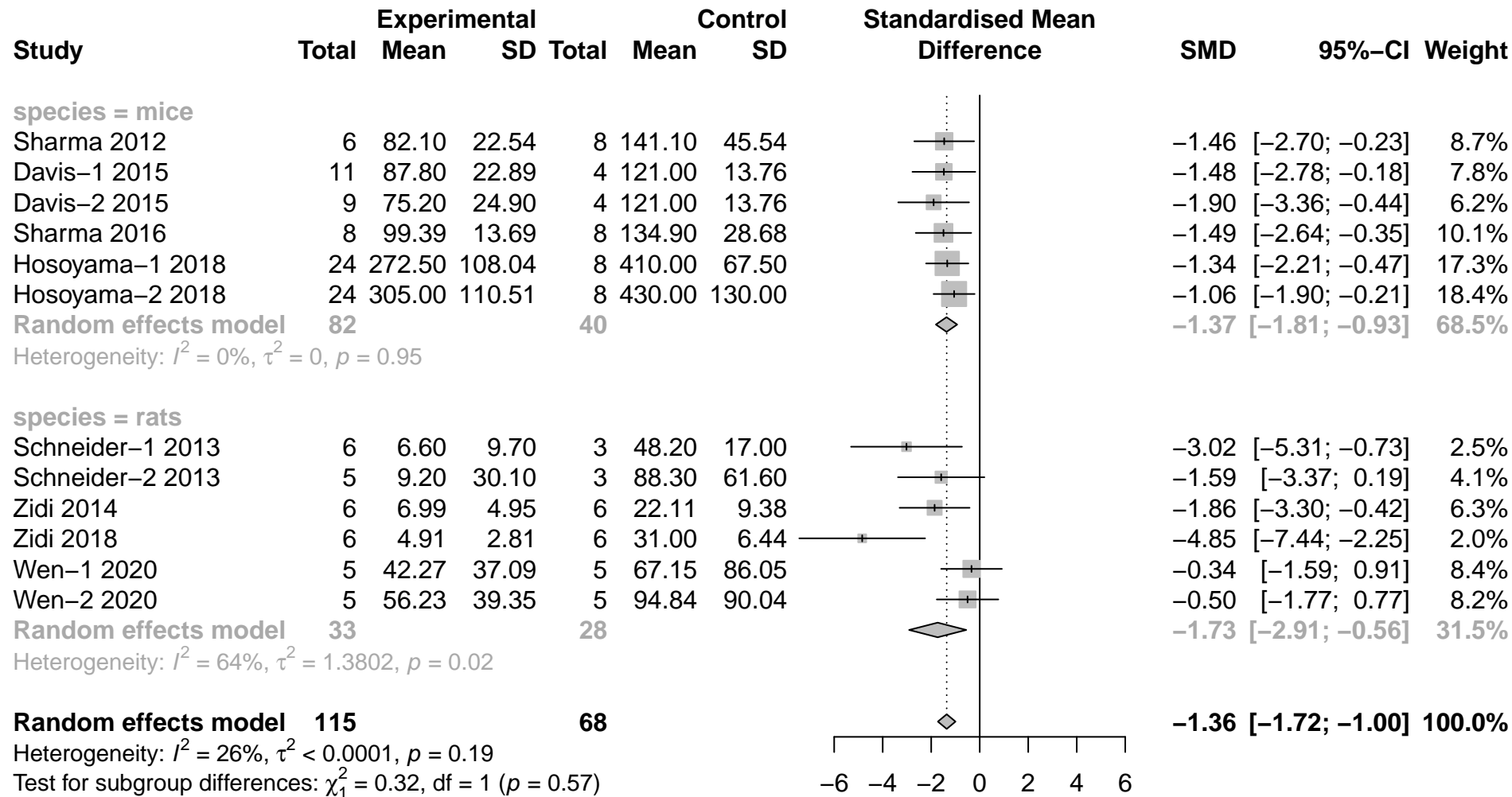

Supplement: Supplementary file 15 — Additional file 15: Fig. S13. Forest plot summarizing the relationship between animal species and diameter change ratio (% increase) in preclinical models of AAA. [file 13287_2022_2755_MOESM15_ESM.pdf]

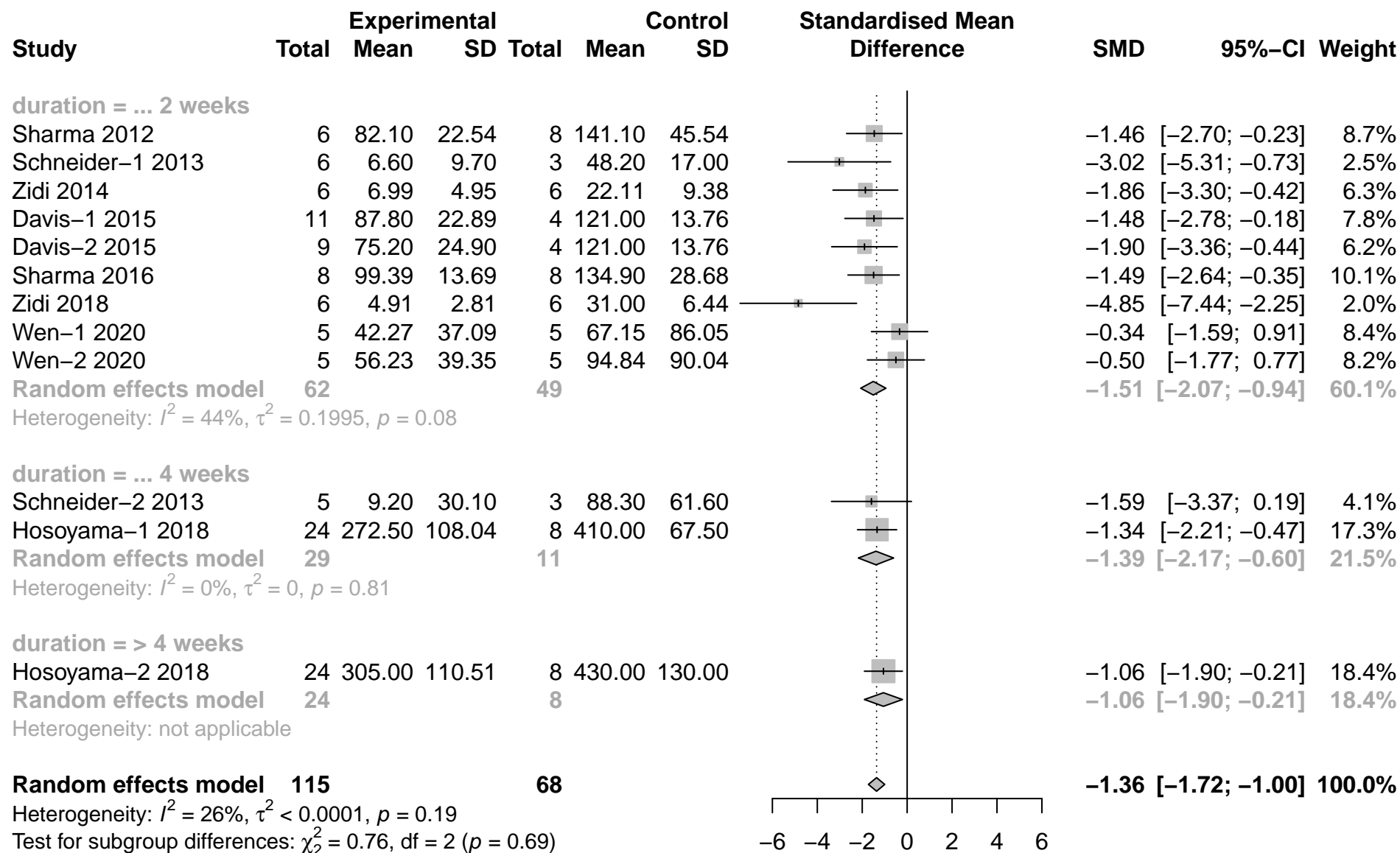

Supplement: Supplementary file 16 — Additional file 16: Fig. S14. Forest plot summarizing the relationship between follow-up duration and diameter change ratio (% increase) in preclinical models of AAA. [file 13287_2022_2755_MOESM16_ESM.pdf]

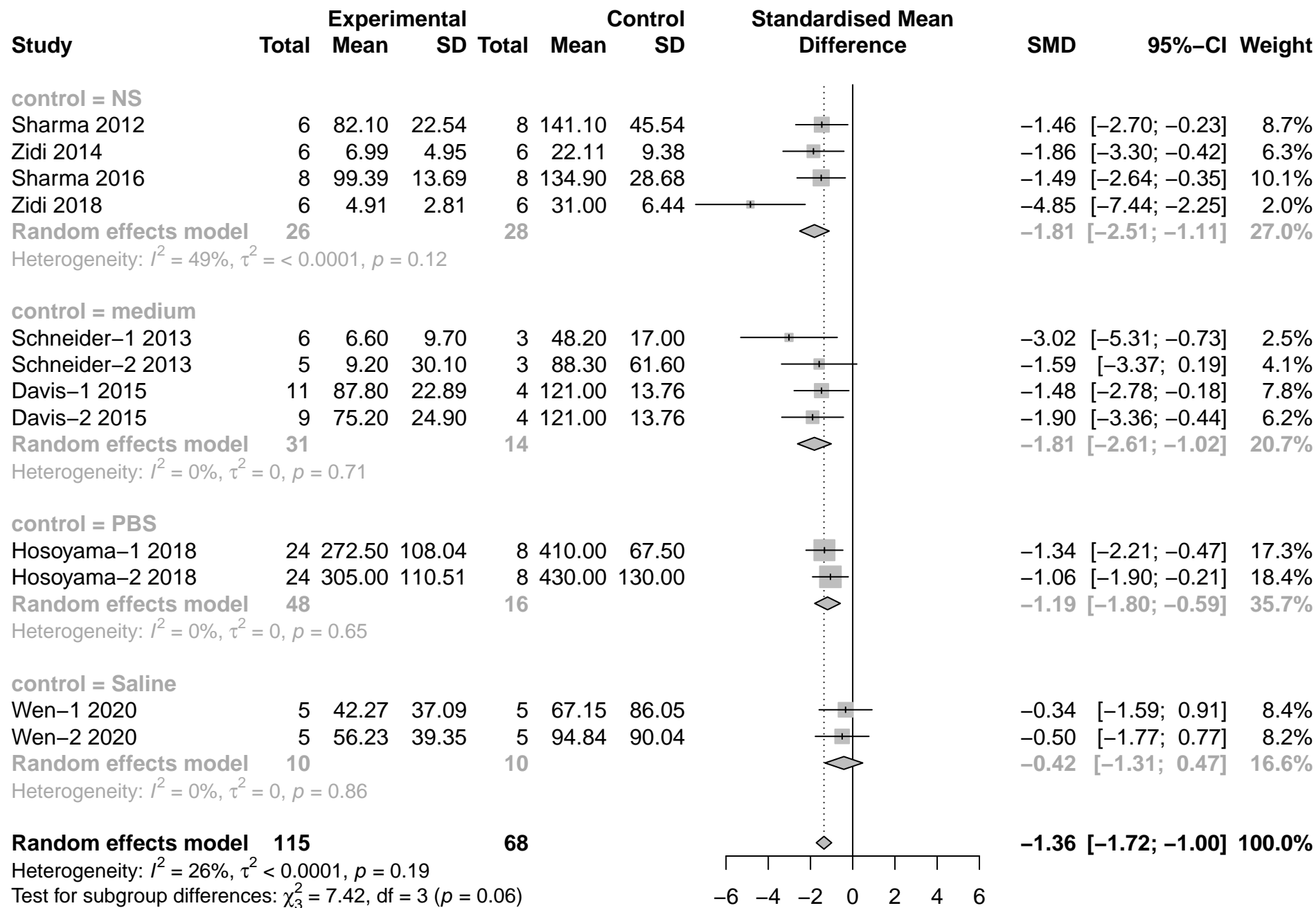

Supplement: Supplementary file 17 — Additional file 17: Fig. S15. Forest plot summarizing the relationship between control type and diameter change ratio (% increase) in preclinical models of AAA. [file 13287_2022_2755_MOESM17_ESM.pdf]

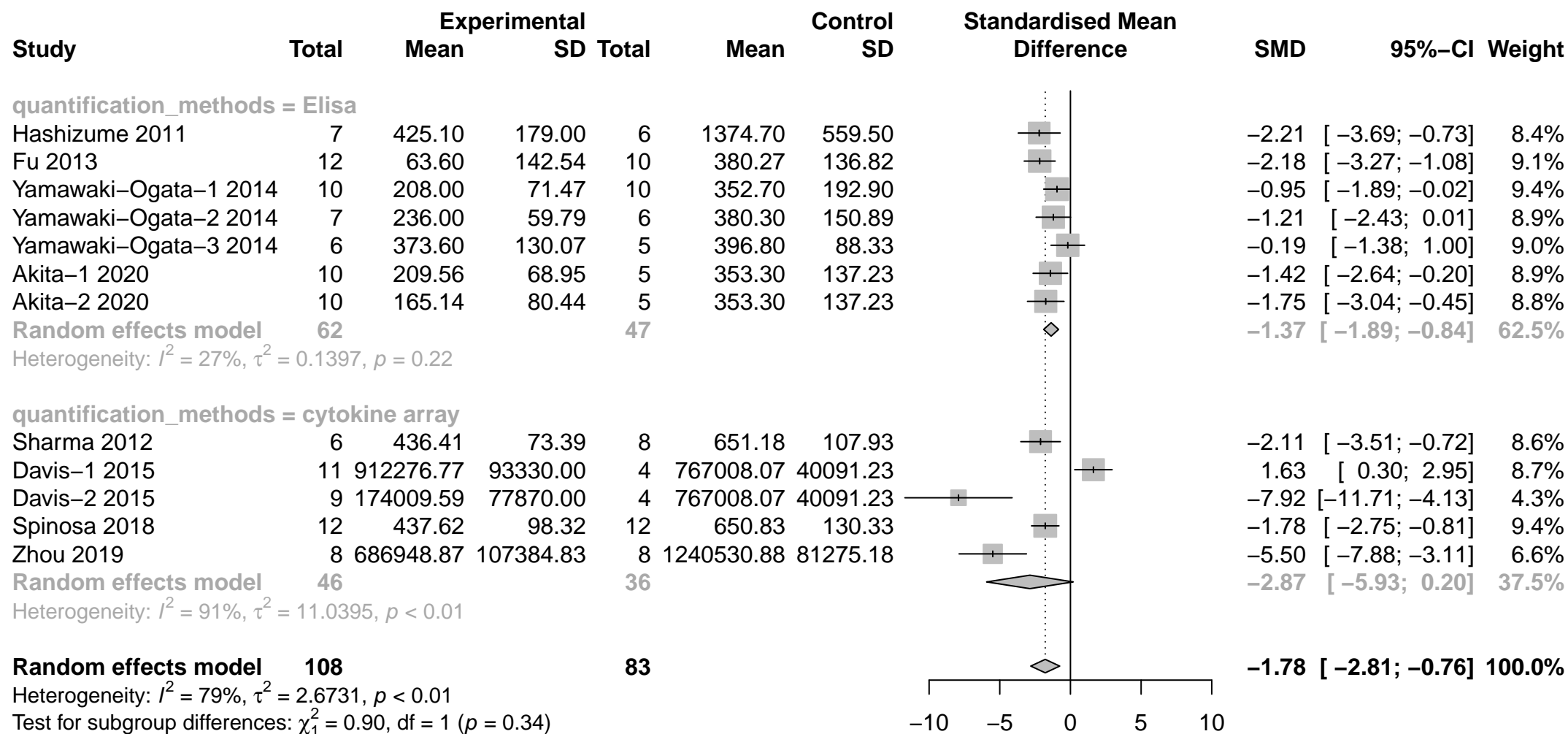

Supplement: Supplementary file 19 — Additional file 19: Fig. S17. Forest plot of the therapeutic effects of MSCs for MCP-1 level in AAA models, compared with control group. [file 13287_2022_2755_MOESM19_ESM.pdf]

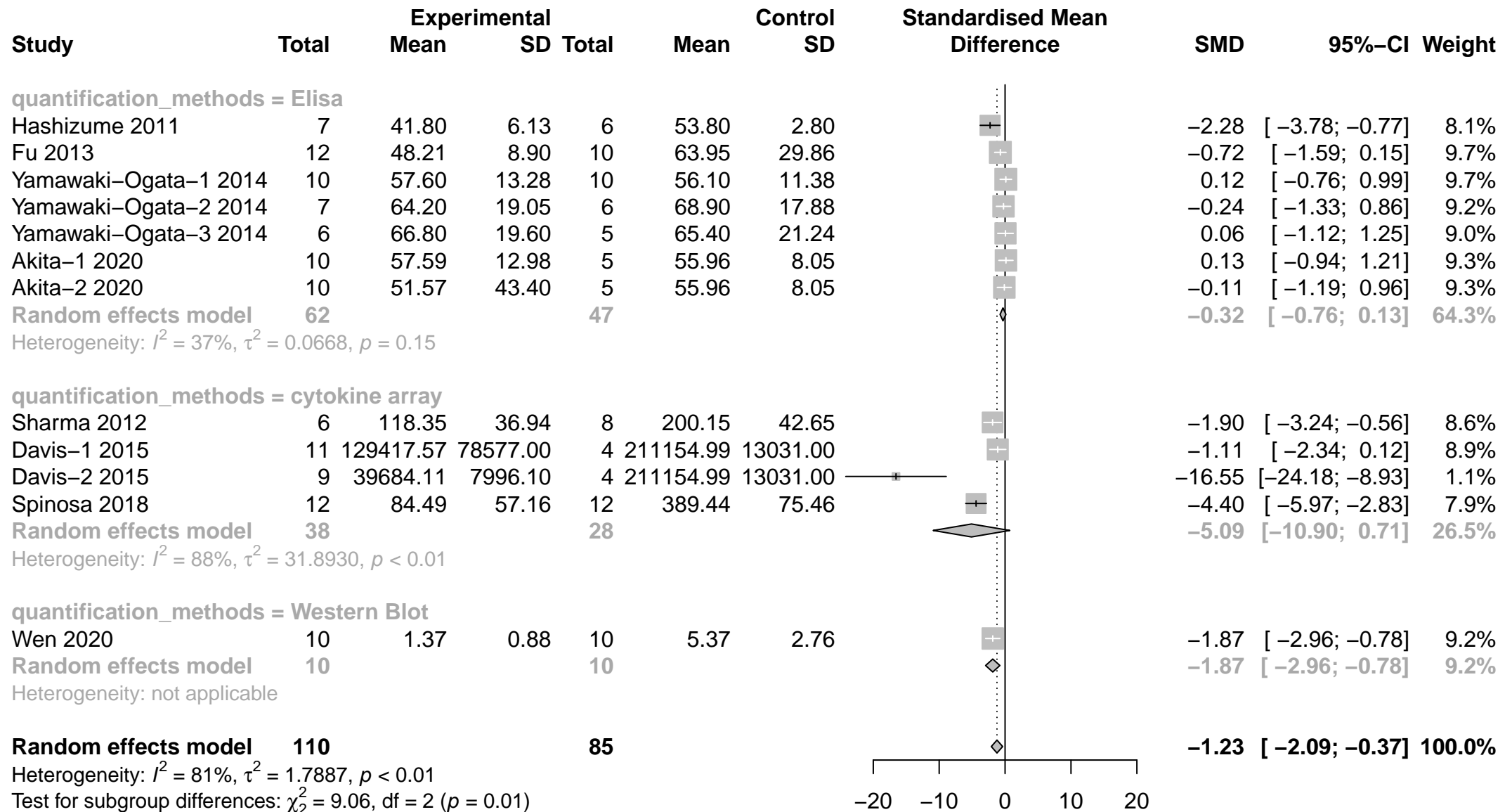

Supplement: Supplementary file 20 — Additional file 20: Fig. S18. Forest plot of the therapeutic effects of MSCs for TNF-α level in AAA models, compared with control group. [file 13287_2022_2755_MOESM20_ESM.pdf]

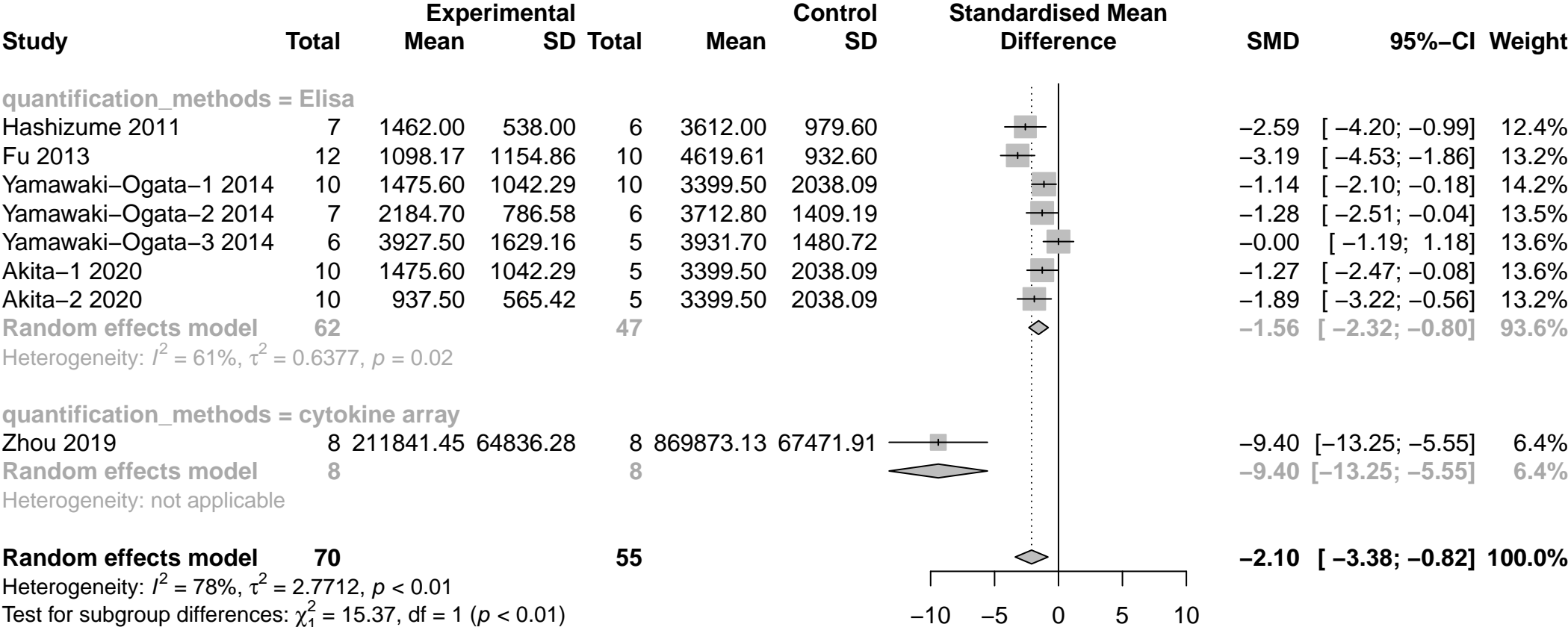

Supplement: Supplementary file 21 — Additional file 21: Fig. S19. Forest plot of the therapeutic effects of MSCs for IL-6 level in AAA models, compared with control group. [file 13287_2022_2755_MOESM21_ESM.pdf]

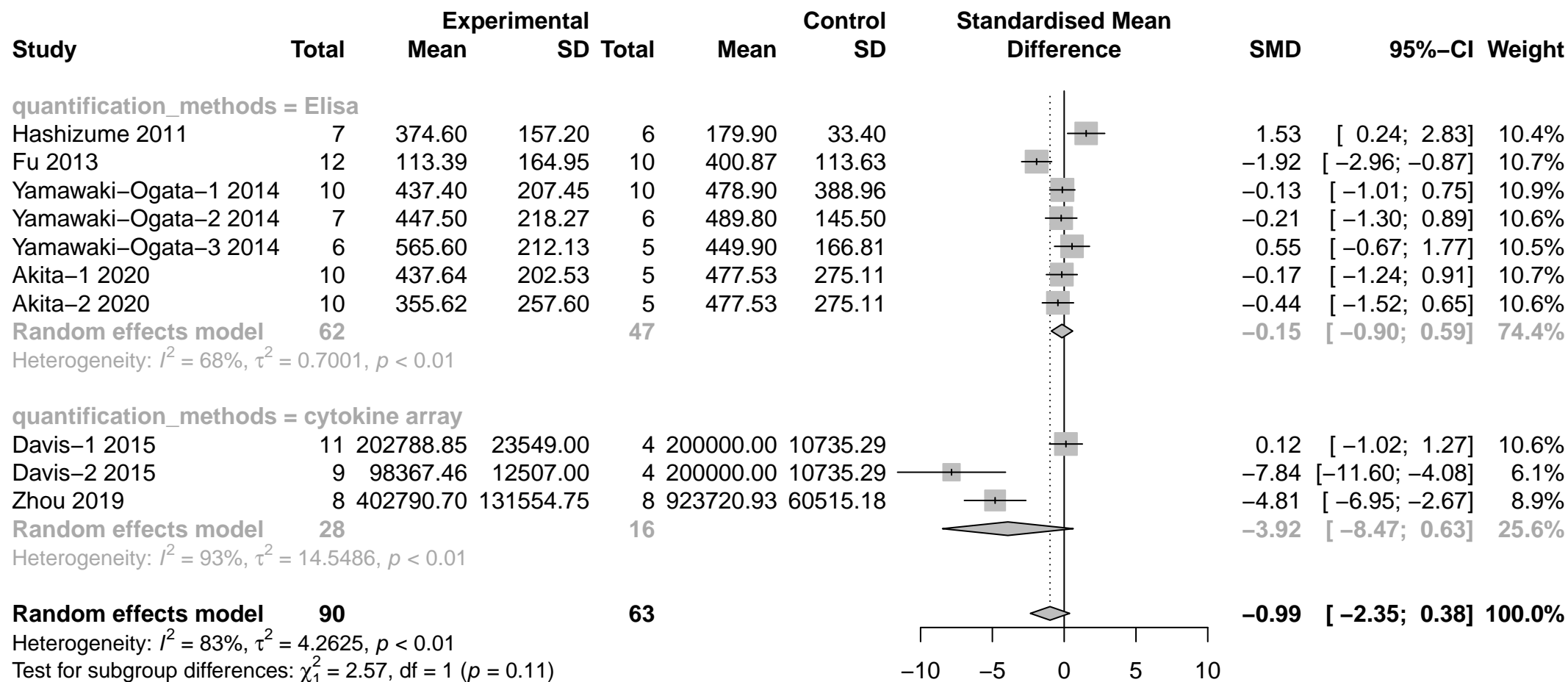

Supplement: Supplementary file 22 — Additional file 22: Fig. S20. Forest plot of the therapeutic effects of MSCs for IL-1β level in AAA models, compared with control group. [file 13287_2022_2755_MOESM22_ESM.pdf]

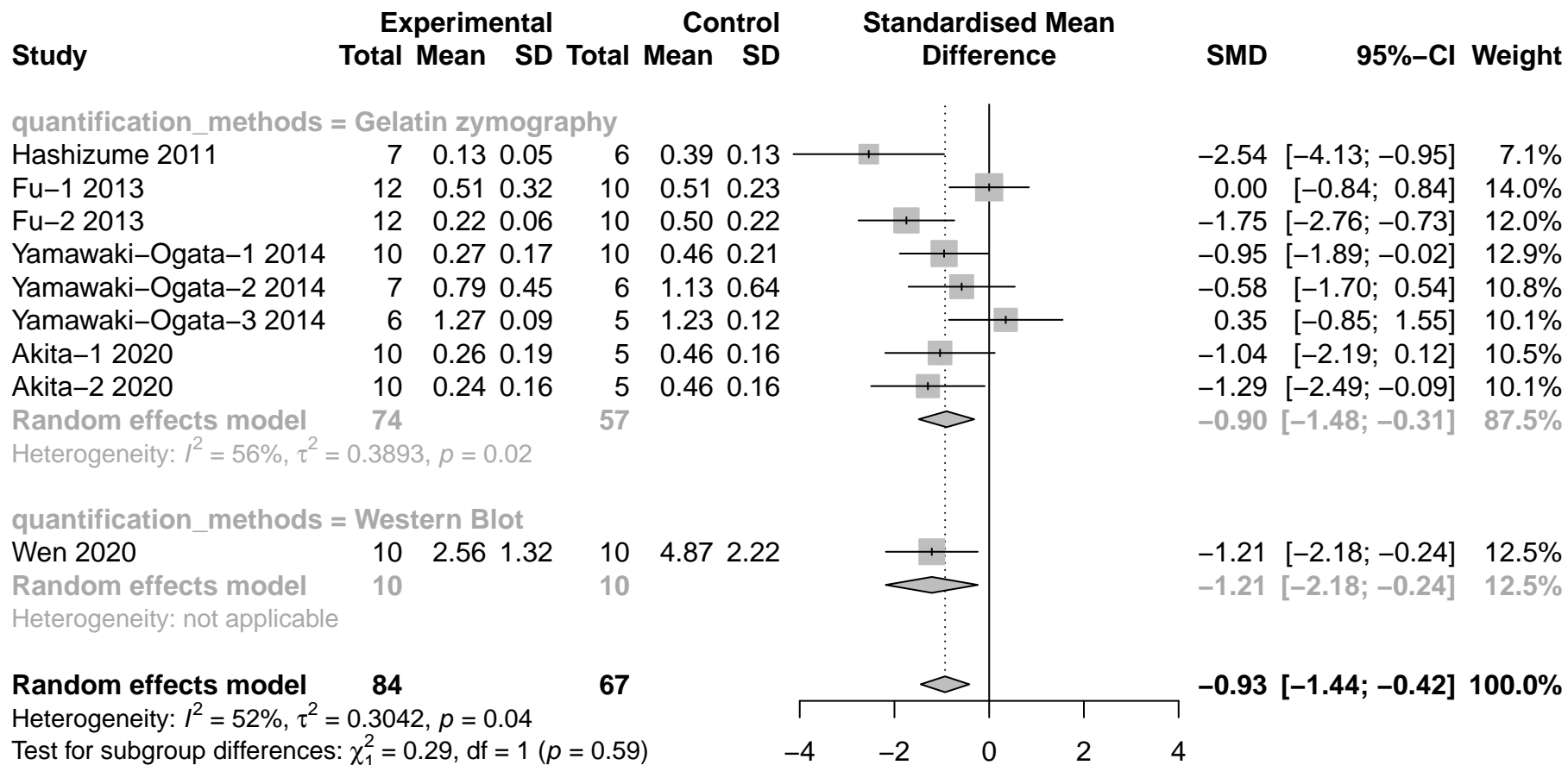

Supplement: Supplementary file 23 — Additional file 23: Fig. S21. Forest plot of the therapeutic effects of MSCs for pro-MMP2 level in AAA models, compared with control group. [file 13287_2022_2755_MOESM23_ESM.pdf]

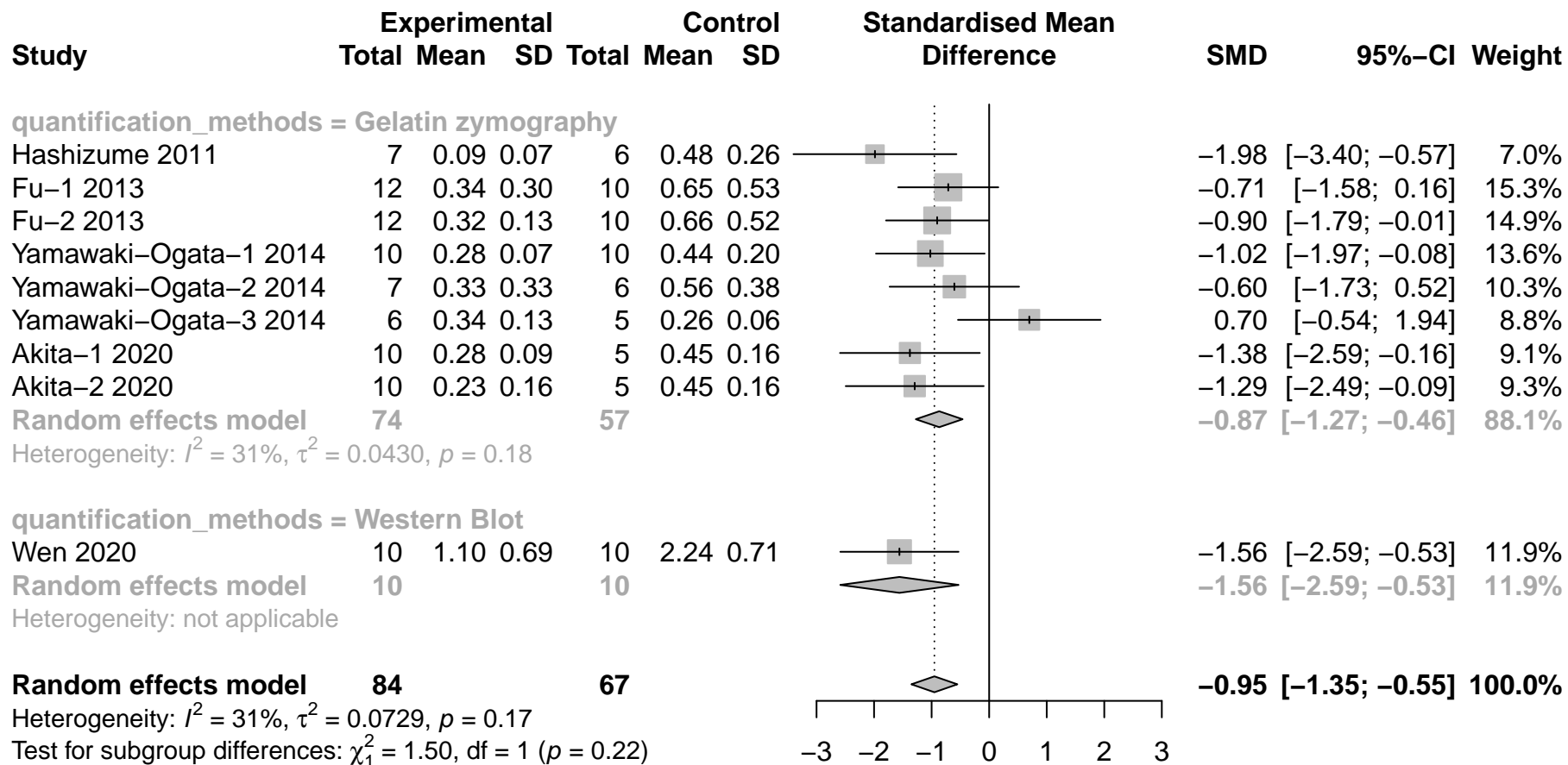

Supplement: Supplementary file 24 — Additional file 24: Fig. S22. Forest plot of the therapeutic effects of MSCs for active-MMP2 level in AAA models, compared with control group. [file 13287_2022_2755_MOESM24_ESM.pdf]

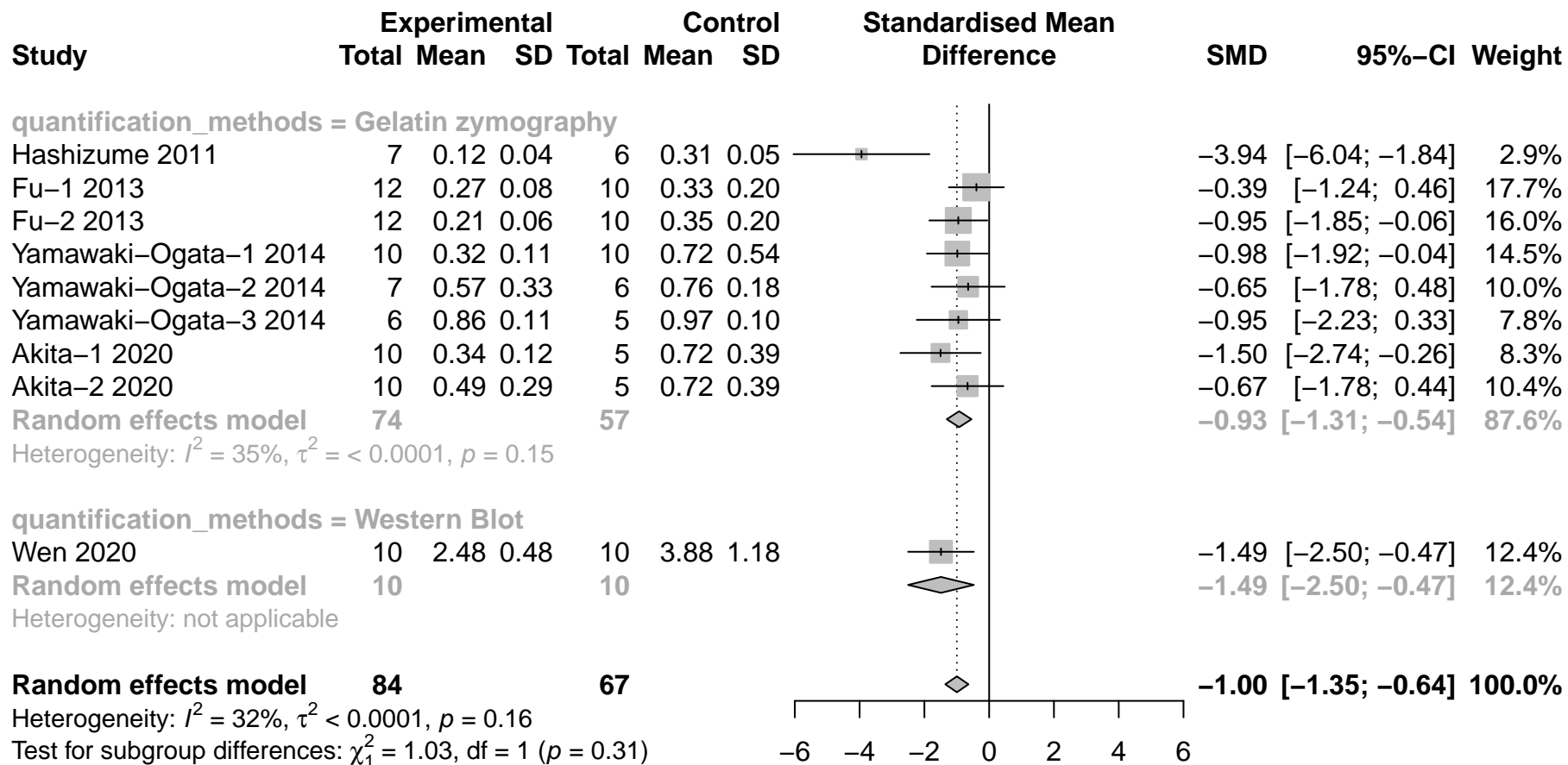

Supplement: Supplementary file 25 — Additional file 25: Fig. S23. Forest plot of the therapeutic effects of MSCs for pro-MMP9 level in AAA models, compared with control group. [file 13287_2022_2755_MOESM25_ESM.pdf]

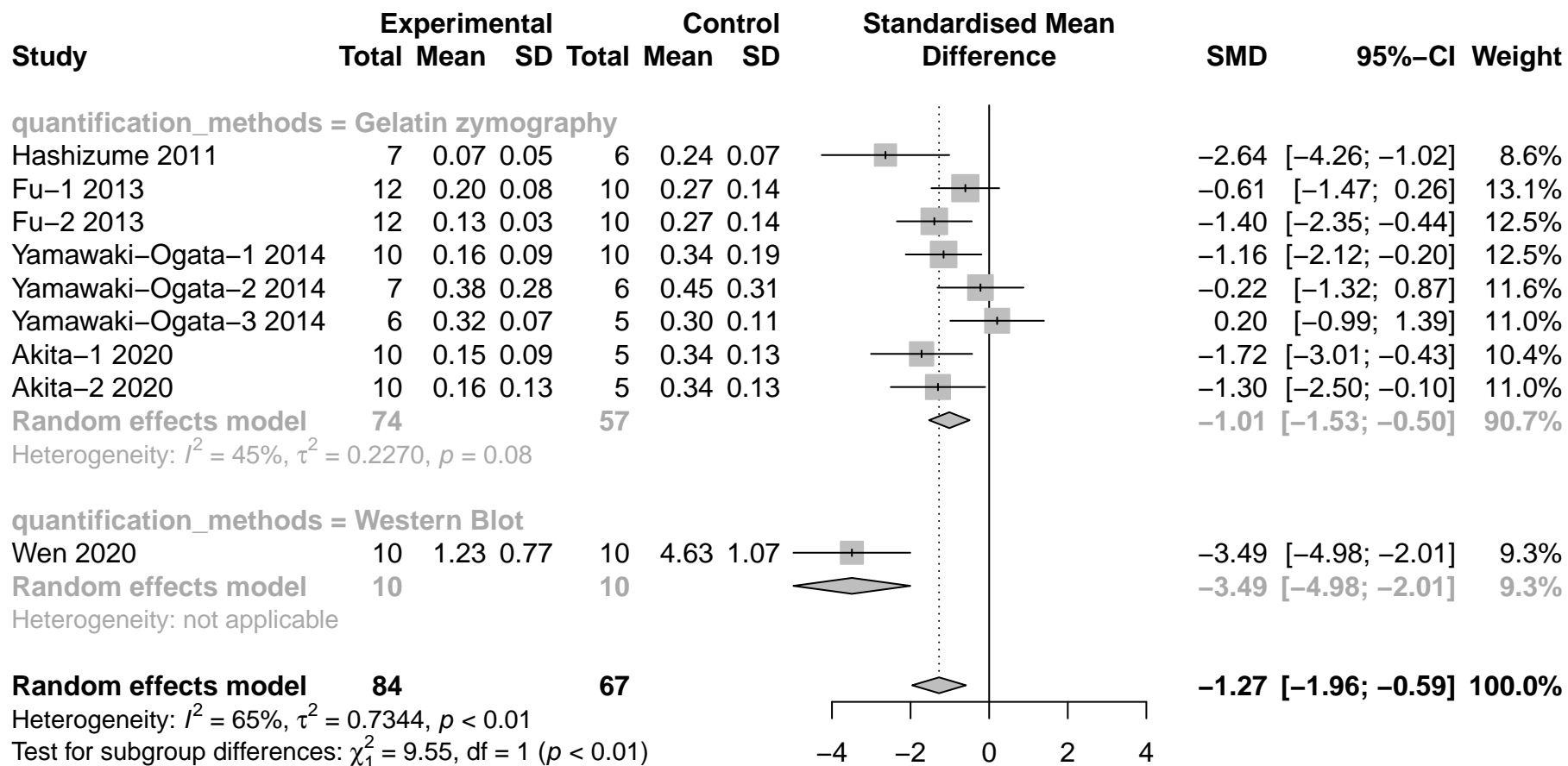

Supplement: Supplementary file 26 — Additional file 26: Fig. S24. Forest plot of the therapeutic effects of MSCs for active-MMP9 level in AAA models, compared with control group. [file 13287_2022_2755_MOESM26_ESM.pdf]

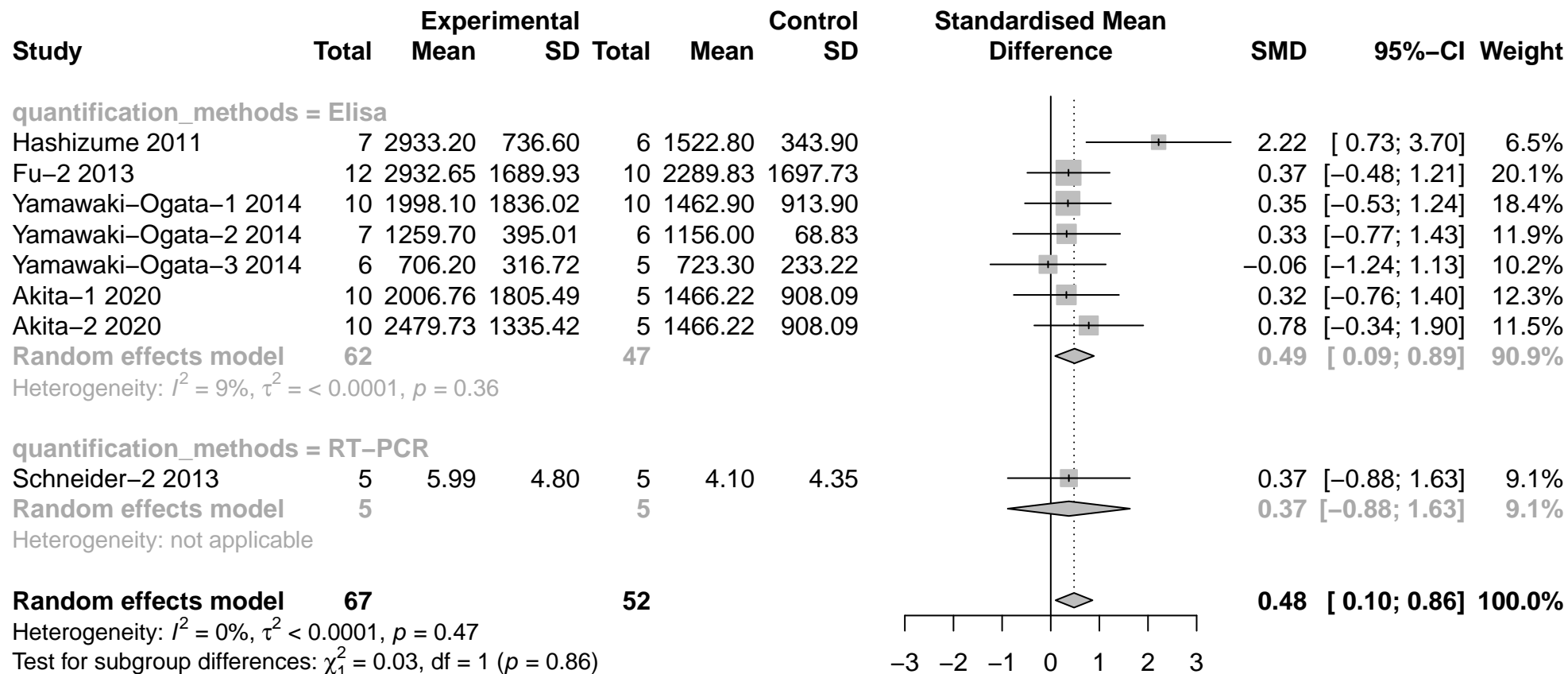

Supplement: Supplementary file 27 — Additional file 27: Fig. S25. Forest plot of the therapeutic effects of MSCs for TIMP-1 level in AAA models, compared with control group. [file 13287_2022_2755_MOESM27_ESM.pdf]
