# Supplementary material for: Therapeutic efficacy of mesenchymal stem cells for abdominal aortic aneurysm: a meta-analysis of preclinical studies
Source: Stem Cell Res Ther. 2022 Feb 24;13:81. doi: 10.1186/s13287-022-02755-w (PMC8867868; doi:10.1186/s13287-022-02755-w)

Study	Experimental Total	Experimental Mean	Experimental SD	Control Total	Control Mean	Control SD	SMD	95%−CI	Weight
quantification_methods = dry weight									
Hashizume 2011	7	46.87	6.22	6	25.48	4.64	3.58	[1.62; 5.54]	3.6%
Fu−1 2013	10	30.33	8.15	5	26.46	9.99	0.42	[−0.67; 1.50]	9.5%
Fu−2 2013	12	39.55	7.55	5	26.22	10.14	1.52	[0.33; 2.71]	8.2%
Yamawaki−Ogata−1 2014	10	30.12	5.35	10	21.39	6.19	1.45	[0.44; 2.45]	10.5%
Yamawaki−Ogata−2 2014	7	30.06	7.63	6	22.00	5.49	1.11	[−0.09; 2.31]	8.1%
Yamawaki−Ogata−3 2014	6	25.03	7.56	5	20.08	7.53	0.60	[−0.63; 1.83]	7.9%
Zhou 2019	8	42.61	6.50	8	25.98	3.47	3.02	[1.47; 4.56]	5.5%
**Random effects model**	**60**			**45**			**1.50**	**[0.75; 2.24]**	**53.4%**
Heterogeneity: $I^2 = 57\%$, $\tau^2 = 0.5696$, $p = 0.03$									
quantification_methods = percent area									
Schneider 2013	5	28.52	16.50	3	4.25	4.30	1.54	[−0.22; 3.30]	4.4%
Hosoyama 2018	24	8.85	4.77	8	5.20	2.10	0.83	[0.00; 1.66]	13.5%
Parvizi 2018	6	1.86	0.53	6	0.71	0.38	2.30	[0.72; 3.89]	5.2%
Akita−1 2020	10	58.40	6.01	5	45.80	7.38	1.83	[0.52; 3.15]	7.1%
Akita−2 2020	10	56.20	6.32	5	45.80	7.38	1.47	[0.24; 2.70]	7.8%
Wen 2020	10	0.81	0.28	5	0.48	0.31	1.07	[−0.09; 2.23]	8.6%
**Random effects model**	**65**			**32**			**1.31**	**[0.82; 1.80]**	**46.6%**
Heterogeneity: $I^2 = 0\%$, $\tau^2 = 0$, $p = 0.59$									
**Random effects model**	**125**			**77**			**1.39**	**[0.99; 1.78]**	**100.0%**

Heterogeneity: $I^2 = 32\%$, $\tau^2 = 0.1194$, $p = 0.12$

Test for subgroup differences: $\chi^2_1 = 0.17$, df = 1 ($p = 0.68$)

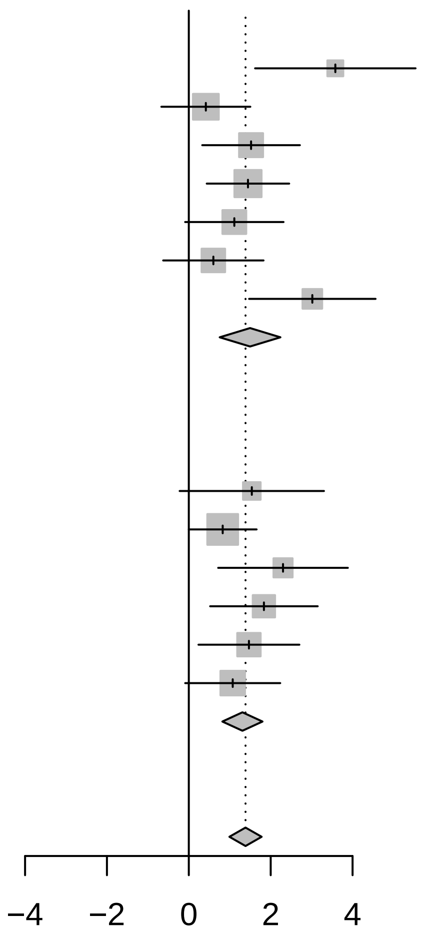

Supplement: Supplementary file 18 — Additional file 18: Fig. S16. Forest plot of the therapeutic effects of MSCs for elastin content in AAA models, compared with control group. [file 13287_2022_2755_MOESM18_ESM.pdf]
