# Supplementary material for: Therapeutic efficacy of mesenchymal stem cells for abdominal aortic aneurysm: a meta-analysis of preclinical studies
Source: Stem Cell Res Ther. 2022 Feb 24;13:81. doi: 10.1186/s13287-022-02755-w (PMC8867868; doi:10.1186/s13287-022-02755-w)
Supplement: Supplementary file 28 — Additional file 28: Fig. S26. Forest plot of the therapeutic effects of MSCs for TIMP-2 level in AAA models, compared with control group. [file 13287_2022_2755_MOESM28_ESM.pdf]

Study	Experimental Total	Experimental Mean	Experimental SD	Control Total	Control Mean	Control SD	Standardised Mean Difference	SMD	95%−CI	Weight
quantification_methods = Elisa										
Hashizume 2011	7	1.60	0.95	6	1.10	0.63		0.57	[−0.55; 1.69]	14.5%
Fu−2 2013	12	41.69	16.36	10	3.93	30.86		1.51	[0.54; 2.49]	17.7%
Yamawaki−Ogata−1 2014	10	9.50	3.16	10	4.00	1.90		2.02	[0.90; 3.14]	14.6%
Yamawaki−Ogata−2 2014	7	5.50	2.38	6	4.50	2.20		0.40	[−0.70; 1.51]	14.8%
Yamawaki−Ogata−3 2014	6	5.30	2.45	5	4.20	2.91		0.38	[−0.83; 1.58]	13.1%
Akita−1 2020	10	9.50	3.16	5	4.00	1.90		1.83	[0.51; 3.14]	11.5%
Akita−2 2020	10	11.10	7.27	5	4.00	1.90		1.09	[−0.08; 2.25]	13.8%
Random effects model	62			47				1.11	[0.61; 1.62]	100.0%
Heterogeneity: I^2 = 27%, τ^2 = 0.1227, p = 0.22										
**Random effects model**	**62**			**47**				**1.11**	**[0.61; 1.62]**	**100.0%**

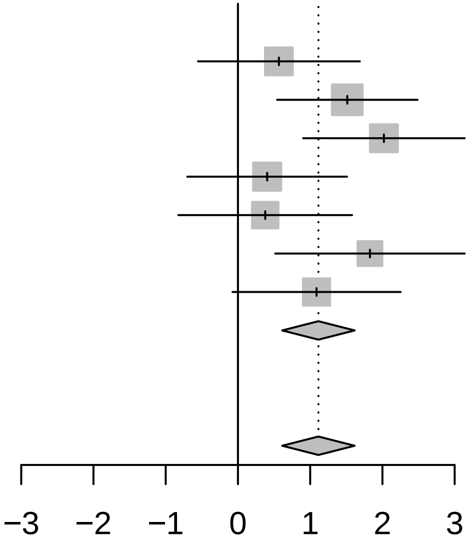

Heterogeneity: I^2 = 27%, τ^2 = 0.1227, p = 0.22

Test for subgroup differences: χ^2_0 = 0.00, df = 0 (p = NA)
